# Recent Advances in Nanomedicine for Ocular Fundus Neovascularization Disease Management

**DOI:** 10.1002/adhm.202304626

**Published:** 2024-03-10

**Authors:** Yifan Zhou, Mingyu Xu, Wenyue Shen, Yufeng Xu, An Shao, Peifang Xu, Ke Yao, Haijie Han, Juan Ye

**Affiliations:** ^1^ Eye Center The Second Affiliated Hospital School of Medicine Zhejiang University Zhejiang Provincial Key Laboratory of Ophthalmology Zhejiang Provincial Clinical Research Center for Eye Diseases Zhejiang Provincial Engineering Institute on Eye Diseases 88 Jiefang Road Hangzhou 310009 P. R. China

**Keywords:** choroidal neovascularization, nanomedicine, ocular drug delivery, retinal neovascularization, smart nanocarriers, stimuli‐responsive

## Abstract

As an indispensable part of the human sensory system, visual acuity may be impaired and even develop into irreversible blindness due to various ocular pathologies. Among ocular diseases, fundus neovascularization diseases (FNDs) are prominent etiologies of visual impairment worldwide. Intravitreal injection of anti‐vascular endothelial growth factor drugs remains the primary therapy but is hurdled by common complications and incomplete potency. To renovate the current therapeutic modalities, nanomedicine emerged as the times required, which is endowed with advanced capabilities, able to fulfill the effective ocular fundus drug delivery and achieve precise drug release control, thus further improving the therapeutic effect. This review provides a comprehensive summary of advances in nanomedicine for FND management from state‐of‐the‐art studies. First, the current therapeutic modalities for FNDs are thoroughly introduced, focusing on the key challenges of ocular fundus drug delivery. Second, nanocarriers are comprehensively reviewed for ocular posterior drug delivery based on the nanostructures: polymer‐based nanocarriers, lipid‐based nanocarriers, and inorganic nanoparticles. Thirdly, the characteristics of the fundus microenvironment, their pathological changes during FNDs, and corresponding strategies for constructing smart nanocarriers are elaborated. Furthermore, the challenges and prospects of nanomedicine for FND management are thoroughly discussed.

## Graphical abstract

Ocular fundus neovascularization diseases are prominent etiologies of visual impairment. Specifically, nanomedicine arose at an opportune time, which has paved the way to fulfill the effective ocular fundus drug delivery and achieve precise drug release control capacity, thus further improving the therapeutic effect. Through advanced synthetic strategies and well‐thought‐out experimental design, certain nanomedicines are endowed with huge potential to progress to the clinic.

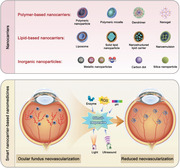

## 1 Introduction

The ocular fundus, as the receptive site for light and the entry point for visual signals, plays a crucial role in visual perception. Angiogenesis, the formation of new blood vessels, is integral to typical developmental progression and physiological functions.^[^ ^1^ ^]^ Nevertheless, the pathological angiogenesis appears in a variety of ocular pathologies, especially fundus neovascularization diseases (FNDs) exemplified by age‐related macular degeneration (AMD), diabetic retinopathy (DR), and retinopathy of prematurity (ROP), which are prominent etiologies of visual impairment worldwide.^[^ ^2^ ^]^ Specifically, AMD, the third leading cause of vision loss globally, is also the first reason for permanent vision impairment in aging individuals in developed nations and areas.^[^ ^3^ ^]^ It is projected that there will be a significant rise in the prevalence of AMD in the coming decades, affecting approximately 288 million individuals by the year 2040.^[^ ^4^ ^]^ DR, the foremost reason for legal blindness among individuals aged 20 to 74 worldwide, ranks among the prevalent microvascular complications linked to diabetes mellitus.^[^ ^5^ ^]^ As per information published by the International Diabetes Federation, it is estimated that by the year 2030, around 210 million people will be affected by DR worldwide,^[^ ^6^ ^]^ indicating that around one‐third of diabetic patients are diagnosed with DR.^[^ ^7^ ^]^ ROP is a proliferative neurovascular disease that impacts prematurely born infants and is recognized for its substantial long‐term implications on vision.^[^ ^8^ ^]^ In the case of preterm births, 73% of infants born with a gestational age of 27 weeks or less are susceptible to developing ROP. Furthermore, the incidence of ROP is on the rise in developed countries, primarily due to the enhanced survival of infants with low birth weights.^[^ ^2^, ^9^ ^]^ Owing to the unique dual blood supply system of the retina, including retinal and choroidal vasculatures, there are two primary types of neovascularization impacting the fundus: choroidal neovascularization (CNV) and retinal neovascularization (RNV), which are severe conditions requiring prompt diagnosis and treatment to prevent irreversible vision loss.^[^ ^10^ ^]^ CNV appears when new vessels grow beneath the retina or within the choroid, which is often associated with AMD.^[^ ^11^ ^]^ Contrarily, RNV appears when new vessels grow on the surface of the retina, which is often associated with DR and ROP.^[^ ^11^, ^12^ ^]^

Although the eye is readily accessible in the body, there exist significant challenges for medication delivered to the ocular posterior segment. Like the brain, the eye is recognized as “immune privileged” due to its status as a crucial component of the central nervous system. It is characterized by sophisticated anatomy featuring complicated ocular barriers, rendering it an exceptionally isolated organ from the systemic circulation. Consequently, treating ocular diseases, particularly in the posterior segment, encounters numerous hurdles. Intravitreal injection (IVT) gained prominence in fundus disease treatment, as it can directly deliver cargo to the ocular posterior segment.^[^ ^13^ ^]^ Fortunately, the advent of drugs targeting vascular endothelial growth factor (VEGF), including pegaptanib (Macugen), bevacizumab (Avastin), ranibizumab (Lucentis), and aflibercept (Eylea), marked an immense breakthrough in the management of FNDs.^[^ ^14^ ^]^ Nevertheless, these VEGF antibodies (anti‐VEGFs) possess several limitations because only a subset of patients (25–40%) gain significant visual improvement.^[^ ^15^ ^]^ Additionally, a substantial number of patients experience an inadequate response to anti‐VEGF injections, identified as 1) durable fluid exudation, 2) unresolved or new hemorrhage, 3) progressive lesion fibrosis, and 4) suboptimal vision recovery.^[^ ^2a^ ^]^ Furthermore, anti‐VEGF is typically required for repeated and lifelong IVTs to maintain its effectiveness, which can cause some short‐term complications, including eye pain, redness, and inflammation.^[^ ^16^ ^]^ In rare cases, it can result in severe enduring complications like macular dysfunction, retinal detachment, and endophthalmitis et al.^[^ ^2^, ^17^ ^]^ In addition to the inherent risks associated with IVT, there are notable systemic risks associated with antibodies exiting the eye, followed by entering the systemic circulation.^[^ ^18^ ^]^ The subtle yet sustained suppression of plasma VEGF levels is hypothesized to be associated with an elevated risk of cardiovascular diseases, including stroke and thromboembolic events.^[^ ^18^, ^19^ ^]^ Moreover, an extensive VEGF depletion in the retina can result in serious adverse effects: the disruption of interactions between the outer segments of photoreceptors and retinal pigment epithelium (RPE) or defects in the RPE‐choroid complex.^[^ ^20^ ^]^

Consequently, there is a compelling need for alternative therapeutic concepts for FNDs. Unfortunately, being a target organ, the eye presents numerous critical internal and superficial barriers that hinder both systemic and direct drug access.^[^ ^21^ ^]^ Thankfully, the recent advances in nanomedicine pave a promising avenue to surmount these barriers. Owing to their ultrafine size, nanocarriers experience substantial changes in crystal configuration and surface electronic structure, manifesting a diverse array of novel functions and properties not observed in their micro‐ or macro‐scale counterparts.^[^ ^22^ ^]^ These nanocarriers possess substantial potential as they offer improved penetration, extended retention, enhanced solubility, reduced toxicity, prolonged drug release, and precise delivery of loaded agents to eyes.^[^ ^23^ ^]^ Crucially, through modification, nanocarriers can effectively tackle the challenges presented by ocular posterior barriers, allowing themselves to meet the demands for efficient ocular posterior drug delivery, thereby enhancing the overall therapeutic efficacy.^[^ ^23c^ ^]^ Furthermore, some nanocarriers, mostly inorganic nanoparticles, possess the dual capability of not only transporting anti‐angiogenic agents but also exerting direct anti‐angiogenic effects, effectively eradicating fundus neovascularization.^[^ ^24^ ^]^ Remarkably, smart nanocarriers have surfaced with the progress of chemistry and materials science.^[^ ^25^ ^]^ Falling within a distinctive category, smart nanocarriers are endowed with specific functionalities responsive to endogenous or exogenous stimuli to precisely release components, contributing to developing innovative nanomedicines gifted with the capacity to display tailored responses to stimulating factors such as light, ultrasound, and biomarkers present in the fundus pathological microenvironment.^[^ ^26^ ^]^ Therefore, smart nanocarriers featuring precise drug release control capacity based on various available stimuli can increase efficacy and reduce side effects to a greater extent, being a rising star in managing FNDs.^[^ ^27^ ^]^

**Figure 1:**
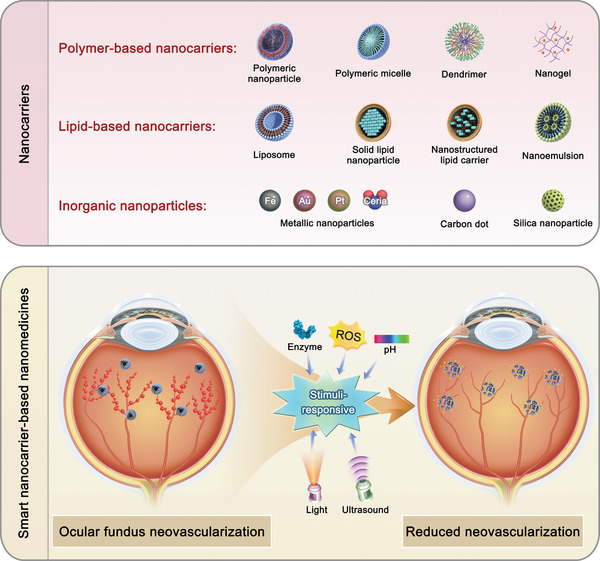
Nanocarriers for ocular posterior drug delivery and smart nanocarrier‐based nanomedicines for FND treatment.

To date, numerous nanomedicines have been developed, demonstrating promising efficiencies in the management of FNDs (**Figure** **1**). Therefore, this review offers a comprehensive overview of the utilization of nanomedicine in ocular posterior drug delivery and the underlying therapeutic strategies of FNDs, thereby offering promising future avenues for the enhancement of treatment and the prognosis of patients with FNDs.

## 2 Current Therapeutic Modalities for FNDs

Owing to its substantial metabolic activity, the retina exhibits the most consumption of oxygen among all organs and tissues.^[^ ^28^ ^]^ When tiny capillaries suffer from damage, as under the circumstances of hyperglycemia in diabetes, the retina quickly experiences hypoxic states, thus leading to neovascularization as a compensatory mechanism for oxygen deficiency. However, the new vessels are often leaky, causing more issues than they resolve. In the sections below, we will briefly recapitulate the ocular fundus anatomy, as well as the pathological characteristics of the three most common FNDs (AMD, DR, and ROP). Moreover, current drug delivery modalities will also be propounded to gain insights into different and standard features of FNDs, the limitations of traditional therapeutic modalities, and the benefits of using nanocarriers.

### 2.1 Ocular Fundus Anatomy

**Figure 2:**
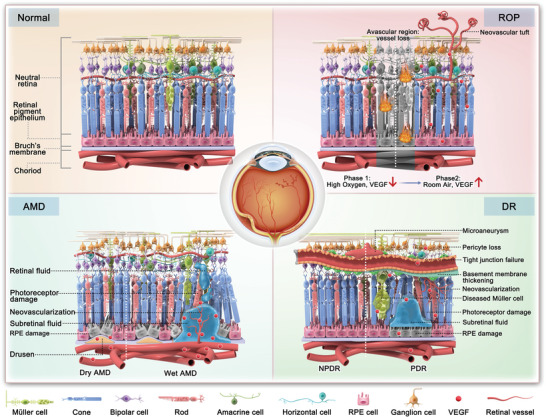
The normal fundus and main pathogenetic events in FNDs (AMD, DR, and ROP).

The retina is composed of diverse cell types, each characterized by unique morphology and function (**Figure** **2**).^[^ ^29^ ^]^ The inner limiting membrane (ILM) constitutes the foremost section of the retina, situated at the vitreo‐retinal interface. The neural retina, situated between the ILM and RPE, encompasses multiple cell types, including retinal ganglion cells, horizontal cells, amacrine cells, bipolar cells, photoreceptor cells, as well as supporting cells like astrocytes, microglia, Müller cells, and retinal vascular endothelial cells (RECs).^[^ ^29^ ^]^ Retinal vessels perform an indispensable role in providing nutrients and oxygen to the inner layers of the retina, excluding photoreceptor cells.^[^ ^30^ ^]^ The rate of blood flow in the retina is notably lower compared to choroidal levels (0.26 mL/h in human retina).^[^ ^31^ ^]^ The RECs mold tight intercellular junctions and comprise the inner component of the blood‐retina barrier (BRB), a protective mechanism shielding the retina from harmful substances.^[^ ^31^, ^32^ ^]^ Pericytes, glial cells, and neurons further contribute to reinforcing this barrier.^[^ ^32^ ^]^

The RPE appears to be a tightly polarized, pigmented, and organized single layer of cells, establishing the outer BRB between the neural retina and the choroid. The apical microvilli of RPE extend towards the photoreceptors, whereas its basal side is oriented toward Bruch's membrane adjacent to the choroid. The RPE's critical functionalities include the sustained phagocytosis of outer segments of cones and rods, facilitating their degradation, as well as the removal of metabolites and fluid, contributing to retinal homeostasis.^[^ ^33^ ^]^ Additionally, it absorbs light, protecting the retina from oxidative damage, and supplies essential nutrients (e.g., fatty acids, retinol, glucose) to the retina.

The choroid is a strongly vascularized layer including tightly pigmented choroidal melanocytes.^[^ ^34^ ^]^ It provides approximately 85% of ocular blood flow (62 mL h^−1^ in humans), providing nutrients and oxygen to the photoreceptors and RPE.^[^ ^35^ ^]^ The fenestrated choroidal capillary walls, with a pore size of 75–85 nm and 46.3± 2.8 pores per square micrometer, facilitate the penetration of macromolecules.^[^ ^36^ ^]^ The innermost section of the choroid, identified as Bruch's membrane, is a fibrous extracellular matrix comprising elastin and collagen. It is fused with the basolateral side of the RPE, forming a relatively weak physical barrier between the choroid and RPE.^[^ ^37^ ^]^

### 2.2 Fundus Neovascularization Diseases

#### 2.2.1 Age‐related Macular Degeneration

AMD is commonly classified into early and late stages (Figure 2). Early AMD is characterized by drusen (≥125 µm), retinal pseudo‐drusen, or pigmentary abnormalities. Late AMD, on the other hand, can be further divided into two forms: 1) Dry AMD, also noted as “non‐neovascular AMD”; 2) Wet AMD, as well noted as “neovascular AMD”.^[^ ^18^ ^]^ Compared to wet AMD, dry AMD has a slower progression and is characterized by the presence of drusen, which are small, yellowish deposits. Over time, this leads to atrophy and thinning of the macula's RPE, choriocapillaris, and photoreceptors.^[^ ^38^ ^]^ While vision loss is less severe in dry AMD patients, a subset of individuals with dry AMD may eventually develop wet AMD.^[^ ^39^ ^]^ Wet AMD is identified by the presence of CNV, leading to bleeding, retinal edema, and eventual fibrosis.^[^ ^40^ ^]^ While only 10–15% of individuals with AMD are affected by the wet form, it is this hemorrhage and exudation into the retina that predominantly leads to over 80% blindness of the overall AMD population. Thus, current research efforts primarily focus on wet AMD.^[^ ^41^ ^]^

#### 2.2.2 Diabetic Retinopathy

DR is characterized by the chronic, progressive deterioration of the neural retina and retinal vessels. Its onset and progression are closely linked to prolonged diabetes, hyperglycemia, and hypertension.^[^ ^7^ ^]^ While traditionally considered a microvascular disease, DR also involves retinal neurodegeneration.^[^ ^7^ ^]^ Like AMD, DR can also be categorized into two forms: non‐proliferative and proliferative forms (Figure 2). Non‐proliferative diabetic retinopathy (NPDR) typically represents the early stage of DR, characterized by the presence of retinal hemorrhages and microaneurysms, which is a consequence of durable hyperglycemia in individuals with diabetes.^[^ ^42^ ^]^ In contrast, proliferative diabetic retinopathy (PDR) is identified as the emergence of neovascularization originating from the retinal venous circulation. These leaky blood vessels penetrate the ILM and extend into the vitreous cavity, which can lead to diabetic macular edema, in which there is exudation and edema in the central part of the retina, and even serious hemorrhage, finally resulting in retinal detachment and the damage to the whole retina.^[^ ^42^, ^43^ ^]^

#### 2.2.3 Retinopathy of Prematurity

Similarly, there are also two phases for the pathogenesis of ROP:^[^ ^44^ ^]^ (Phase 1) Initially, there is a delay in the normal growth of retinal vessels, leading to an avascular region in the peripheral retina. (Phase 2) Subsequently, there is a significant proliferation of blood vessels, manifesting as intravitreal angiogenesis, which can occur at the boundary between the vascularized and avascular regions of the retina. The increased VEGF, induced by hypoxia as depicted in Figure 2, disrupts the orderly development of retinal blood vessels, thereby delaying the natural vascular development process. Conversely, reduced VEGF levels in high‐oxygen environments also hinder the physiological development of retinal blood vessels by diminishing developmental angiogenesis. Once the disease occurs, the progression is rapid, and the window for effective treatment is narrow. Therefore, premature infants born before 37 weeks of gestation should be examined promptly, and high‐risk individuals should be inspected weekly.

Despite varying etiologies, FNDs share typical pathophysiological neovascularization, which precipitates a sequence of events contributing to vision loss: Initial fluid and hemorrhage accumulation due to these new vessels in the retina, eventually escalate into retinal detachment, followed by the degeneration or death of photoreceptor cells, ultimately leading to visual impairment.^[^ ^11^ ^]^ In the healthy eye, the formation of new vessels is tightly controlled by a delicate equilibrium between anti‐angiogenic and pro‐angiogenic factors. Typically, the equilibrium tends to favor an anti‐angiogenic state due to the prevalence of anti‐angiogenic factors.^[^ ^45^ ^]^ Retinal hypoxia or oxidative stress in the RPE and outer retina can push this delicate equilibrium towards pro‐angiogenic, which can induce the upregulation of hypoxia‐inducible factor 1 (HIF‐1), initiating a signaling cascade causing the overproduction of VEGF, the most significant pro‐angiogenic factor, along with upregulating several other pro‐angiogenic factors.^[^ ^11^, ^45^ ^]^ When this happens, the equilibrium is based on neovascularization. In FNDs, visual impairment often arises from the pathological processes of excessive neovascularization and aberrant modification of existing vasculature.^[^ ^1^ ^]^

### 2.3 Ocular Fundus Drug Delivery Barriers Affecting Drug Delivery Modalities

Delivery of medications to the fundus is challenging despite the eye being easily accessible, which necessitates addressing four key challenges: 1) traversing multiple barriers; 2) targeting specific fundus cell types; 3) delivering applicable therapeutic cargos; 4) achieving durable drug release over an extended time.^[^ ^46^ ^]^ IVT of anti‐VEGF remains the first‐line therapy for FNDs. The main challenge in the therapy lies in extending the intervals between IVTs, ensuring a sufficient concentration of medication stays in the vitreous for an adequate duration.^[^ ^47^ ^]^ Therefore, maintaining effective drug concentrations can only be achieved by frequent injections, significantly contributing to the incidence of side effects and placing a burden on patients. Therefore, understanding the local drug delivery barriers of the fundus and the defects of current drug delivery administrations, thus developing nanomedicine delivery platforms, can be optimized to enhance the anti‐angiogenic treatment effectively.

#### 2.3.1 Ocular Fundus Drug Delivery Barriers

On the whole, the drug delivery physiological barriers to the fundus can be broadly categorized into three major groups: 1) ILM, which limits the penetration of intravitreal agents into the fundus; 2) retinal endothelium (inner BRB), which limits the access of xenobiotics from the bloodstream into the retina; 3) RPE (outer BRB), which restrains the inward and outward transferring of molecules between the choroid and the neural retina.

The ILM is an acellular membrane characterized by a negative charge and primarily composed of polysaccharides, including glycosaminoglycans,^[^ ^48^ ^]^ which appears to be denser with a pore size of ≈10 nm in comparison to the mesh size of the vitreous (≈500 nm),^[^ ^49^ ^]^ theoretically allowing for the passage of biologics from the vitreous into the fundus.^[^ ^50^ ^]^ Notably, the detectable penetrations of polylactide nanoparticles (140 nm, 300 nm) and liposomes (50 nm) from the vitreous into the fundus were demonstrated.^[^ ^51^ ^]^ However, it appears plausible that the factors beyond particle size may also influence the permeation of agents through the ILM.

Endothelial tight junctions of retinal capillaries permit restricted paracellular permeation within the inner BRB only. Transporters in the inner BRB contribute to the selectivity of permeation for hydrophilic molecules, ions, and water to enter or exit the retina, while small lipophilic molecules are capable of diffusing through the inner BRB.^[^ ^52^ ^]^

The RPE, with its intercellular tight junctions, establishes the outer BRB, separating the choroid from the retina. A pioneering study demonstrated that microperoxidase (mean diameter: 2 nm) was incapable of permeating through the RPE.^[^ ^53^ ^]^ Subsequently, it was demonstrated that molecular diffusion through the RPE is determined by the size and lipophilicity of the molecules.^[^ ^54^ ^]^ In bovine RPE ex‐vivo, hydrophilic fluorescein isothiocyanate isomer I (FITC)‐dextran (with a mean molecular weight of 77 kDa) displayed RPE permeability 35 and 318‐fold lower compared to that of lipophilic (betaxolol) and hydrophilic (carboxyfluorescein) small molecules, respectively.^[^ ^55^ ^]^ Lipophilic betaxolol penetrated eight‐fold faster than hydrophilic atenolol through ex vivo rabbit RPE‐choroid specimens.^[^ ^54b^ ^]^ In a similar study, a substantial range of approximately 2000‐fold in mean outward RPE permeability values was observed in ex vivo bovine RPE‐choroid specimens (e.g., ketorolac at 69 × 10^−6^ cm s^−1^; bevacizumab at 0.035 × 10^−6^ cm s^−1^).^[^ ^54c^ ^]^

**Figure 3:**
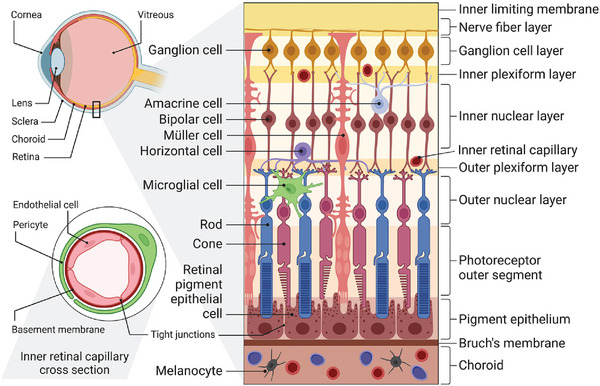
Schematic presentation of the main anatomy of the ocular fundus. The ILM and BRB represent crucial barriers restricting the fundus drug delivery. The BRB is composed of two primary parts: the inner BRB, characterized by tight junctions between RECs, and the outer BRB, formed by tight junctions between RPE cells. Reproduced under the terms of the Creative Commons Attribution license 4.0 (CC‐BY) (https://creativecommons.org/licenses/by/4.0/).^[^ ^29^ ^]^ Copyright 2023, The Authors, published by Elsevier.

The inner BRB and the outer BRB together compose the whole BRB (**Figure** **3**), which effectively hinders the passage of drugs through systemic administration.^[^ ^46^ ^]^ Small molecule drugs have the capability of permeating across both the inner BRB and the outer BRB, which typically results in their elimination from the vitreous to the bloodstream across these barriers.^[^ ^31^, ^50^ ^]^ Consequently, the majority of small molecule drugs injected intravitreally tend to distribute to the retina before ultimately exiting the eye. In contrast, protein drugs are primarily cleared via anterior routes, such as aqueous humor outflow.^[^ ^56^ ^]^ This means that protein drugs primarily permeate into the neural retina, and just a few injected doses manage to traverse the BRB and finally enter the systemic circulation.

Overall, numerous potential drugs for fundus diseases often encounter obstacles in reaching therapeutic concentrations at their intended target sites. This predicament emphasizes the urgency to explore alternative methods of drug delivery and therapeutic strategies capable of circumventing the protective mechanism posed by these barriers.

#### 2.3.2 Ocular Posterior Drug Delivery Modalities

**Figure 4:**
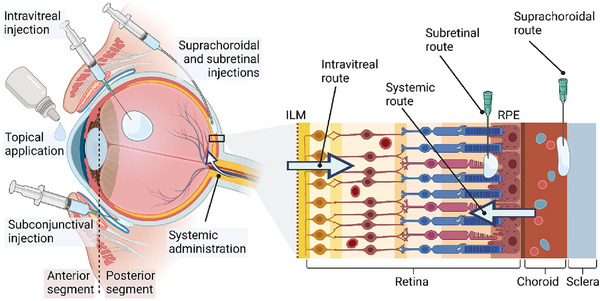
Schematic presentation of ocular posterior drug delivery administration routes. Reproduced under the terms of the CC‐BY.^[^ ^29^ ^]^ Copyright 2023, The Authors, published by Elsevier.

Drug delivery to the posterior segment of the eye presents challenges because drugs must traverse multiple barriers. Drugs applied topically exhibit minimal bioavailability to the fundus, as a significant portion of the administered drug either flushes away from the ocular surface or is absorbed into the systemic circulation. Additionally, the epithelium on the ocular surface imposes significant limitations on drug permeation (**Figure** **4**).^[^ ^57^ ^]^ Typically, only 0.07–4.3% doses of the small molecule drugs reach the ocular anterior segment,^[^ ^58^ ^]^ and only a negligible fraction of drugs is further distributed to the ocular posterior segment because the fundus drug delivery barriers and removal processes associated with flow eliminate the major fraction of the drug reaching the ocular anterior segment.^[^ ^59^ ^]^ As for the systemic administration, the distribution of drugs to the retina from the systemic circulation is constrained by the BRB and the binding of drugs with proteins present in the plasma.^[^ ^60^ ^]^ Even with sub‐conjunctival injections, less than 1% of the doses reach the vitreous and retina (Figure 4).^[^ ^61^ ^]^ Therefore, IVT is commonly chosen for drug delivery to the fundus, especially in the management of FNDs (Figure 4). Extending the intervals between IVTs could be advantageous for both patients and healthcare professionals. To illustrate this, researchers have been investigating controlled‐release systems aimed at prolonging these intervals, such as implants. Nonetheless, these systems deliver drugs to the retina without specific cell targeting or specificity.^[^ ^47^ ^]^ Suprachoroidal and subretinal injections have the potential to bring medications in close proximity to the retina, although infrequently employed. However, these methods are more intricate and demanding compared to the widely accepted IVTs, and additionally, the interconnections between the photoreceptors and Müller cell endfeet significantly constrain the inward penetration of molecules (Figure 4).^[^ ^62^ ^]^

## 3 Nanocarriers for Ocular Posterior Drug Delivery

A successful drug delivery system (DDS) is defined by ideal biocompatibility, stability, delivery efficiency, biodegradability, and appropriate clearance time. The development of nanocarriers presents these advantages, along with encompassing the overcoming of ocular barriers, enhancement of transcorneal permeability, extension of drug residence time, reduction in dosing frequency, improvement of patient compliance, mitigation of drug degradation, achievement of sustained/controlled release, and facilitation of drug targeting and gene delivery.^[^ ^63^ ^]^ Therefore, nanocarriers represent the most promising avenue with the potential to address these key challenges, which are currently in the developmental stages and emerging in an endless stream.

Nanocarriers with various structures, such as polymer‐based nanocarriers, lipid‐based nanocarriers, and inorganic nanoparticles, have demonstrated promising biochemical properties in both vivo and vitro studies for ocular applications.^[^ ^64^ ^]^ Subsequently, we systematically elaborate on advancements in various nanocarriers for ocular posterior drug delivery, where the key point is in FND management.

### 3.1 Polymer‐Based Nanocarriers

Polymer‐based nanocarriers possess a polymer‐based structure that allows effective encapsulation of drugs, resulting in reduced dissolution and diffusion of the drug. They possess remarkable versatility, allowing precise control over their characteristics and facilitating ease of surface modification.^[^ ^65^ ^]^ According to different nanostructures, polymer‐based nanocarriers can be classified into polymeric nanoparticles, polymeric micelles, dendrimers, and nanogels.^[^ ^23c^ ^]^

#### 3.1.1 Polymeric Nanoparticles

Polymeric nanoparticles are composed of natural polymers (e.g., hyaluronic acid (HA), chitosan, alginate, albumin, collagen, and gelatin) and synthetic polymers (e.g., polylactic acid (PLA), poly(ε‐caprolactone) (PCL), polyethyleneimine (PEI), and poly(lactic‐co‐glycolic acid) (PLGA)). These polymers were noted for their outstanding biocompatibility and biodegradability. The transition in the physicomechanical properties of polymeric nanoparticles can be achieved by finely tuning the composition, cross‐linking, molecular weight, and interactions with other components of the polymer. This precise modulation aims to optimize the performance of injectables in the ocular space, mitigate the risk of bio‐contamination, and enhance the stability of the carriers.^[^ ^66^ ^]^

**Figure 5:**
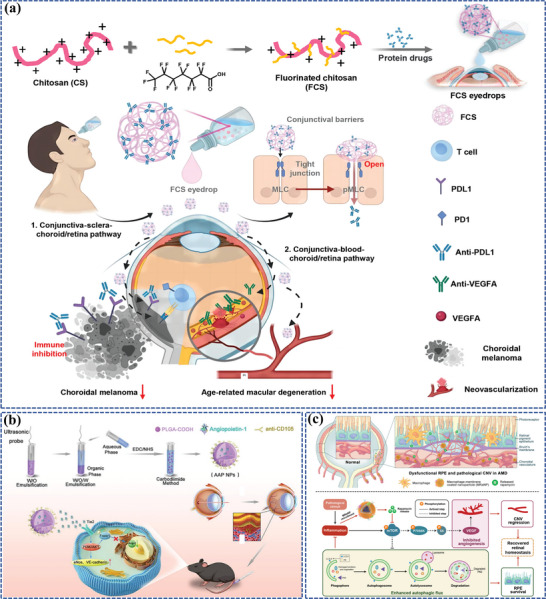
Polymer‐based nanocarriers for ocular posterior drug delivery: a) Schematic illustration of FCS as the nanocarrier for penetrating ocular barriers and enabling effective delivery of macromolecules for treating fundus diseases. Reproduced under the terms of the Creative Commons Attribution Non‐Commercial License 4.0 (CC BY‐NC) (https://creativecommons.org/licenses/by‐nc/4.0/).^[^ ^76^ ^]^ Copyright 2023, American Association for the Advancement of Science. b) The preparation process of biodegradable AAP NPs and AAP NPs targeted long‐term noninvasive treatment of CNV. Reproduced with permission.^[^ ^77^ ^]^ Copyright 2023, Elsevier. c) Schematic diagrams of MRaNPs targeting CNV via the intravenous route and modulating retinal homeostasis through the mTOR signaling pathway. Reproduced with permission.^[^ ^78^ ^]^ Copyright 2022, Elsevier.

Natural polymers possess distinct advantages such as cell‐activated proteolytic degradation, bioactivity, improved membrane permeability, and minimal to no toxicity.^[^ ^67^ ^]^ Among these, HA, a biodegradable biopolymer that occurs naturally in the vitreous body of the eye, possesses excellent biocompatibility and non‐immunogenicity when used in biomedical applications.^[^ ^68^ ^]^ Moreover, HA contains ligands for receptors, including CD44, which are present in retinal cells, contributing greatly to its utility in delivering drugs to the eyes. As an illustration, Radwan et al. developed apatinib‐loaded bovine serum albumin nanoparticles coated with HA (Apa‐HA‐BSA‐NPs), where the HA coating prevented adhesive buildup in the outflow pathway and enabled precise delivery of apatinib, which is a novel anti‐VEGF with the potential to treat DR, to the targeted lesion by binding to CD44 selectively.^[^ ^68^ ^]^ Similarly, Huang et al. developed HA‐coated albumin nanoparticles (HSA NPs) encapsulating a Connexin43 mimetic peptide (Cx43MP), which plays a role in improving inflammation and vessel leakage by inhibiting unregulated hemichannel open.^[^ ^69^ ^]^ Moreover, HA is now considered to have the potential to develop nanocarriers to load small interfering ribonucleic acid (siRNA) to employ RNA interference, which involves the specific recognition of target genes through base pairing with a complementary sequence, followed by the degradation of the target genes by intracellular nucleases.^[^ ^70^ ^]^ Trimethyl chitosan‐HA nanoparticles represent a promising platform for delivering VEGF receptor (VEGFR) siRNA, with HA contributing to enhanced transfection efficiency and reduced degradation rate, while chitosan ensures good biocompatibility.^[^ ^71^ ^]^ Chitosan contains diverse functional groups, including carboxyl, amino, and hydroxyl groups, which interact with mucosal surfaces through hydrogen bond formation, providing mucoadhesion properties.^[^ ^72^ ^]^ Due to the mucoadhesive property, chitosan‐based nanoparticles can significantly prolong their retention in ocular physiological environment, impeding drug clearance.^[^ ^73^ ^]^ In a clinical trial (NCT03192137), DexaSite, a combination of dexamethasone and crosslinked polyacrylic acid and chitosan polymers, has demonstrated favorable therapeutic effects in alleviating inflammation and pain post‐ocular surgery, as the improved viscosity for effective dexamethasone delivery is primarily attributed to the addition of chitosan. Besides serving as a drug carrier, chitosan possesses inherent anti‐angiogenic capabilities. Zahir et al. developed thiolated chitosan nanoparticles, which exhibited increased mucoadhesion ability due to the presence of the thiol group, leading to longer retention time and enhanced therapeutic effects, having the potential to prevent the formation of neovascularization.^[^ ^74^ ^]^ Moreover, chitosan is recognized as a non‐viral promising nanoplatform for gene delivery due to its advantages, including high transfection efficacy, low immunogenicity, and the absence of mutational possibilities when compared to virus vectors.^[^ ^75^ ^]^ A representative example of chitosan‐based nanocarriers is an eyedrop‐based macromolecular ocular DDS (**Figure** **5**a).^[^ ^76^ ^]^ In this study, fluorocarbon‐modified chitosan (FCS) was synthesized through grafting perfluoroalkyl carboxylic acid to chitosan via amide coupling. Such FCS was able to create a stable nanoparticle complex where therapeutic macromolecular proteins self‐assemble through static interactions, temporarily loosening the tight connections between corneal and conjunctival tissues for enhanced ocular penetration, facilitating the delivery of anti‐VEGF and other macromolecular drugs. Encouragingly, in models of CNV in mice and rabbits, FCS combined with the anti‐VEGF manifested significant treatment efficacy compared to intravitreal anti‐VEGF injections. Furthermore, FCS combined with the programmed death ligand 1 antibody (anti‐PDL1) enhanced the anti‐tumor response in choroidal melanoma mice. This innovative design thus introduces an effective delivery carrier for the ocular posterior segment through therapeutic protein eye drops.

Polyester nanoparticles, unlike natural polymers, are synthetic polymers with notable stability, tunability, reproducibility, biocompatibility, and biodegradability in vivo, making them promising solutions for producing nanocarriers.^[^ ^79^ ^]^ PLGA nanoparticle, recognized as one of the least biodegradable and toxic synthetic polymers, has excellent capacities for drug loading, delivery, and sustained release of a wide range of cargo molecules, including both hydrophobic and hydrophilic small molecules as well as large biopharmaceuticals,^[^ ^80^ ^]^ which have been studied to load with Cx43MP to deliver to the retina,^[^ ^81^ ^]^ interleukin‐12 (IL‐12) to down‐regulate MMP9 and VEGF for DR treatment,^[^ ^82^ ^]^ bevacizumab to target pathological angiogenesis,^[^ ^83^ ^]^ the soluble extracellular domain of the very low‐density lipoprotein receptor (VLN‐NP) to target Wnt signaling pathway to treat RNV,^[^ ^84^ ^]^ and cytotoxic criflavine (an inhibitor of HIF‐1 and HIF‐2) to provide long‐term suppression of CNV with sustained drug‐release properties from the suprachoroidal space.^[^ ^85^ ^]^ Studies have disclosed that PLGA nanoparticles loaded with fenofibrate and pioglitazone, respectively, are also efficient treatments for AMD and DR.^[^ ^86^ ^]^ Intriguingly, Bao et al. reported exosome‐loaded degradable PLGA nanoparticles with micrometric pores, where PLGA helped induce a mild self‐healing process during which the superficial surface pores healed, creating specific “ExoCap” pseudo cells and then contributed to sustainedly releasing therapeutic exosomes as the nanoparticles degraded, to treat vitreoretinal diseases.^[^ ^87^ ^]^ Moreover, further modified PLGA nanoparticles could fulfill the co‐delivery of multiple drugs targeting different pathogenic factors, which has been recognized as a promising strategy, offering advantages over monotherapy in tackling multifactorial diseases. To this end, electrostatically conjugated bevacizumab‐bearing PLGA/PEI nanoparticles loaded with dexamethasone (aBev‐DPPNs) were developed, where the inclusion of PEI, extensively employed in DDSs because of its proton sponge effect,^[^ ^88^ ^]^ facilitated the absorption of bevacizumab.^[^ ^89^ ^]^ In both in vitro and in vivo studies, aBev‐DPPNs exhibited impressive anti‐angiogenic therapeutic effects for potential intravitreal applications. Moreover, the same team further modified aBev‐DPPNs with arginine‐glycine‐aspartic acid (RGD), a specific ligand targeted RPE cells and the CNV region, named aBev/cRGD‐DPPNs, which showed higher efficiency of cell uptake and anti‐angiogenic effect.^[^ ^90^ ^]^ Besides, a noninvasive targeted long‐term therapeutic strategy for CNV has been designed by Yao et al., named angiopoietin1‐anti CD105‐PLGA nanoparticles (AAP NPs) (Figure 5b).^[^ ^77^ ^]^ The AAP NPs, with PLGA as the nanocarrier, released angiopoietin 1 (ang1) slowly and targeted the CD105 marker on CNV to increase drug accumulation, which also enhanced cadherin expression between vascular endothelial cells, significantly reducing neovascularization and inhibiting angiopoietin 2 (ang2) secretion by endothelial cells.

Intriguingly, Li et al. innovatively engineered hybrid cell membrane‐coated PLGA nanoparticles, combining retinal endothelium cell (REC) and red blood cell (RBC) membranes to block VEGF effectively.^[^ ^91^ ^]^ The REC membrane coating played a significant therapeutic role by acting as a “nanosponge,” absorbing VEGF and preventing its binding to VEGFR, resulting in decreased leakage and CNV area. Additionally, the homotypic targeting property of REC membrane and the immune evasion capability of RBC membrane contributed to augmenting accumulation in the retina. Likewise, Xia et al. adopted macrophage cell membranes to coat PLGA nanoparticles, followed by loading rapamycin, the typical mechanistic target of rapamycin (mTOR) signaling pathway inhibitor with promising therapeutic potential for AMD by suppressing inflammation, enhancing dysregulated autophagy, and inhibiting angiogenesis downstream of VEGF, to treat CNV intravenously (Figure 5c).^[^ ^78^ ^]^ The macrophage‐coated nanoparticles (MRaNPs) successfully traversed the impaired BRB, improving rapamycin's bioavailability and resulting in anti‐angiogenic effects, reduced inflammation, and activated autophagy in the mice models. These research have paved the avenue for future clinic studies to assess the cell membranes‐coated PLGA nanoparticles applied in human patients, offering a promising alternative to enhance ocular posterior drug delivery and manage FNDs.

Not only drug delivery but PLGA nanoparticles were used for gene delivery. Zhang et al. developed PLGA nanoparticles encapsulating a HIF‐1α short hairpin RNA (shRNA) plasmid, showing promising potential to treat CNV.^[^ ^92^ ^]^ In another study, PLGA nanoparticles modified with RGD can effectively deliver microRNA‐539‐5p, which is found to be overexpressed in the area of CNV and acts as a regulator of CXC‐chemokine receptor 7 (CXCR7), to pathological tissues, including the CNV area.^[^ ^93^ ^]^

However, certain inherent drawbacks, such as non‐uniform particle sizes, initial burst release, protein instability, and potential inflammatory response, have been identified, which can limit their delivery efficacy and biocompatibility of PLGA nanoparticles.^[^ ^94^ ^]^ To prolong the release profile and enhance the delivery efficacy, chitosan has been used as a component to combine with PLGA. For instance, chitosan‐coated PLGA nanoparticles were employed to enable effective sub‐conjunctival administration of bevacizumab, enhancing permeability through the electrostatic interaction between the negatively charged ocular surface and the positively charged chitosan.^[^ ^95^ ^]^ Furthermore, chitosan‐N‐acetyl‐l‐cysteine (CNAC), a chitosan derivative combined with N‐acetyl‐l‐cysteine (NAC), has demonstrated even more potent mucoadhesive properties through the formation of strong disulfide bonds with the cysteine‐rich regions of mucus glycoproteins, further enhanced by NAC. This enhanced mucoadhesion capability facilitates better retention of nanoparticles in ocular tissues. To prevent the exposure of proteins to potentially harmful organic solvents or the use of sonication in the nanoparticle fabrication process, researchers have adopted a pressure quench and supercritical infusion technology. This approach was utilized to create porous micro‐sized PLGA nanoparticles, effectively encapsulating bevacizumab‐coated PLA nanoparticles and facilitating the sustained release of bevacizumab for four months.^[^ ^96^ ^]^ Aimed to mitigate the issue of acidic environments generated during the biodegradation of PLGA into glycolic acid and lactic acid, where acidic conditions can accelerate drug release and degrade protein drugs, researchers helped prevent the rapid decrease in pH by blending PLGA with poly(cyclohexane‐1,4‐diyl acetone dimethylene ketal) (PCADK), a polyketal derived from the synthesis of two neutral compounds (2,2‐dimethoxypropane and 1,4‐cyclohexanedimethanol), thereby stabilizing protein drugs and ensuring a more controlled drug release profile.^[^ ^97^ ^]^ Furthermore, incorporating albumin into the DDS provides an alternative method for steadying protein drugs.^[^ ^98^ ^]^ Besides, chitosan‐modified PLGA nanoparticles loaded with rapamycin were also formulated to harness their anti‐oxidative and anti‐inflammatory properties for AMD treatment.^[^ ^98^ ^]^

**Figure 6:**
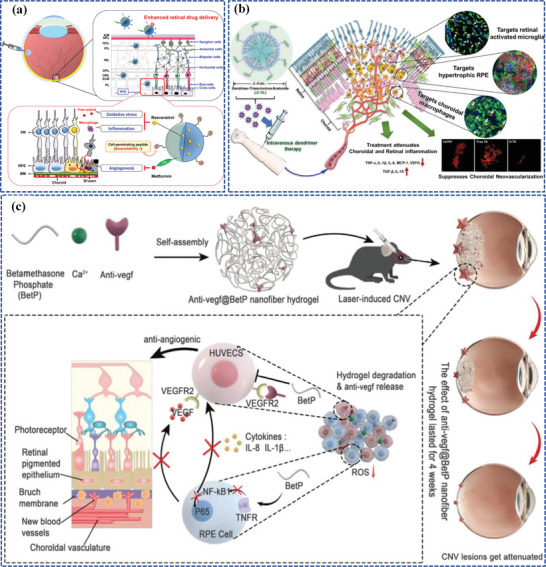
Polymer‐based nanocarriers for ocular posterior drug delivery. a) The pharmacological treatment of wet AMD utilizing R@PCL‐T/M aims to concurrently mitigate oxidative stress, inflammation, and angiogenesis. Reproduced with permission.^[^ ^100^ ^]^ Copyright 2023, American Chemical Society. b) The dendrimer‐triamcinolone acetonide conjugate exhibits promising potential in ameliorating CNV due to its unique macrophage/RPE targeting and anti‐inflammatory properties. Reproduced with permission.^[^ ^101^ ^]^ Copyright 2021, Elsevier. c) The preparation of the Anti‐VEGF@BetP nanofiber hydrogel and its mechanism for treating CNV. Reproduced with permission.^[^ ^102^ ^]^ Copyright 2023, Wiley‐VCH.

Apart from PLGA nanoparticles, PLA and PCL nanoparticles were also utilized to manage FNDs. For instance, the therapeutic potential of retinylamine‐loaded PLA nanoparticles as a promising therapy for AMD, even Stargardt disease, has been demonstrated.^[^ ^99^ ^]^ Additionally, given that PCL is FDA‐approved, facilitating its use in clinical trials, Nguyen et al. present a novel nanotherapeutic (**Figure** **6**a) that combines high retinal permeability with durable bioactive drug delivery, R@PCL‐T/M, which is designed by aminolysis of resveratrol‐encapsulated PCL nanoparticles, followed by the formation of amide linkages with carboxyl‐terminated transacting activator of transcription cell‐penetrating peptides and metformin, where PCL is considered preferred over PLGA and PLA for RPE regions due to its higher biocompatibility and less acidic degraded by‐products.^[^ ^100^ ^]^ In animal models, a single‐dose IVT of R@PCL‐T/M significantly enhances retinal penetration (approximately fifteen‐fold surge), preserves endogenous antioxidants, and suppresses abnormal RNV in cases of macular degeneration over a span of fifty‐six days, which demonstrates significant promise in developing pharmacological nanoformulations that can effectively target the retina, leading to improved treatment outcomes for complex posterior segment diseases.

Polydopamine (PDA) nanoparticles also have shown promise in fundus drug delivery. To provide a sustained and complete treatment of AMD, Allyn et al. adopted heme‐albumin, which can induce heme oxygenase‐1 (HO‐1) to provide anti‐inflammatory protection in RPE cells, integrated into PDA nanoparticles, contributing to heme‐albumin sustainedly release for up to six months, even with quicker release observed under higher oxidative stress conditions.^[^ ^103^ ^]^ Intriguingly, an innovative melanin‐compensatory therapeutic strategy for AMD was developed recently.^[^ ^104^ ^]^ Melanin is naturally present in RPE cells to help counteract oxidative stress, but its antioxidant capacities deplete with the aging process. Therefore, to mimic and replenish the melanin to counteract oxidative stress, Kwon et al. prepared polyethylene glycol (PEG)‐modified synthetic melanin‐like nanoparticles (MNPs) by oxidative polymerization of dopamine hydrochloride in the presence of sodium hydroxide with slight modifications, offering an alternative and promising therapeutic strategy for safeguarding the RPE and photoreceptors, further treating AMD.

#### 3.1.2 Polymeric Micelles

Polymeric micelles are colloidal dispersions with highly organized core–shell supramolecular structures ranging in size from 10 to 200 nm. These micelles are derived from amphiphilic block or graft copolymers that can self‐assemble spontaneously in aqueous solutions when their concentrations exceed the critical micellar concentration.^[^ ^105^ ^]^

Polymeric micelles have been engineered in order to improve corneal penetration as well as facilitate ocular posterior drug delivery. PEG‐PCL micelles to transport hydrophobic dasatinib for intravitreal administration to effectively inhibit RPE cell adhesion, proliferation, and migration, were reported.^[^ ^106^ ^]^ Intriguingly, the anti‐Flt1 peptide‐HA conjugate micelle, where anti‐Flt1 peptide (Gly‐Asn‐Gln‐Trp‐Phe‐Ile, GNQWFI) is an antagonistic hexa‐peptide for VEGF receptor 1 (VEGFR1 or Flt1) and inhibits the binding of VEGF and VEGF/PIGF heterodimer to Flt1,^[^ ^107^ ^]^ has been established in addressing fundus neovascularization, and demonstrated an outcome blocking VEGF dose‐dependently.^[^ ^108^ ^]^ Kim et al. went a step further and sought to incorporate genistein, a small molecule exerting antioxidant and anti‐inflammatory properties, into the same micelle to achieve a synergistic effect in reducing vessel leakage associated with DR.^[^ ^109^ ^]^ Mandal et al. designed micellar formulations of lipid prodrug cyclic cidofovir, formulated through solvent evaporation followed by film rehydration, for targeted retinal delivery, but the study was only conducted in vitro.^[^ ^110^ ^]^ Based on a similar synthesis method, Gote et al. prepared self‐assembling tacrolimus micelles with a polymeric mixture of PEG‐hydrogenated castor oil‐40 (HCO‐40) and octyxonyl‐40 (OC‐40) as the outer layer and tacrolimus as the core, exerting antioxidant and anti‐inflammatory capacities to treat AMD.^[^ ^111^ ^]^ Surprisingly, micelles are capable of topically delivering cargo to the ocular posterior segment, which is exceptionally challenging. In vivo, investigations of chitosan oligosaccharide‐valylvaline‐stearic acid micelles have demonstrated their remarkable capacity to reach the posterior segments via the conjunctival route.^[^ ^112^ ^]^ Encouragingly, Zhao et al. devised a micelle‐based nanoplatform using copolymer EPC (nEPCs), which comprises PCL, PEG, and poly(propylene glycol) (PPG) components, for topically delivering aflibercept to treat FNDs. In this research, nEPCs loaded with aflibercept were able to penetrate the cornea in porcine eye ex vivo models, demonstrating biocompatibility both in vitro and in vivo, delivering a clinically sufficient quantity of aflibercept to the retina in CNV murine models, and causing CNV regression. Moreover, nEPCs demonstrated inherent anti‐angiogenic capabilities, resulting in synergistic outcomes with VEGF agonists.^[^ ^113^ ^]^

#### 3.1.3 Dendrimers

Dendrimers are highly branched and uniform polymers with nanoscale sizes, well‐defined structures, and abundant functional end groups.^[^ ^114^ ^]^ As effective ocular DDSs, dendrimers offer advantages such as enhanced biodistribution, improved drug solubility, increased ocular permeation, and prolonged drug release.^[^ ^115^ ^]^ The widely utilized dendrimers comprise poly(amidoamine) (PAMAM) dendrimers, phosphorus‐containing dendrimers, and poly‐l‐lysine dendrimers.^[^ ^116^ ^]^ Surface modifications, such as PEGylation or acetylation, can be applied to improve the biosafety and carrier ability of these dendrimers.^[^ ^117^ ^]^ Furthermore, with their multifunctional cores and well‐defined nanostructure, dendrimers can act as the fundamental units in the formation of nanogels or liposomes, which possess large internal hydrophobic pores, enabling them to exhibit excellent loading capacity for ocular drug delivery. For example, Wu et al. devised a nanoglobular dendrimer adopting a conjugate of 5‐aminosalicylic acid (5‐ASA), an FDA‐approved agent that has recently demonstrated effectiveness in treating retinal degeneration in animal models, binding a hydrolyzable Schiff base spacer, which exhibited superior efficacy compared to free 5‐ASA as well as a prolonged release, making it a potential treatment option for retinal degeneration.^[^ ^118^ ^]^

Currently, PAMAM‐derived dendrimers stand out as the most widely utilized and commercially available ocular DDSs. They offer several advantages, including enhanced drug bioavailability, more favorable biological response, and improved tolerability. Moreover, these dendrimers help reduce drug clearance from the body, particularly when administered via sub‐conjunctival injection.^[^ ^101^, ^119^ ^]^ For instance, both systemic and intravitreal dendrimers have been specifically directed toward activated microglia.^[^ ^120^ ^]^ Kambhampati et al. observed dendrimers conjugated with cyanine 5‐dye were phagocytized by activated retinal macrophages/microglia (ma/mi) in a disease mouse model, leading to an extended residence time.^[^ ^120^ ^]^ These dendrimers remained detectable for up to 21 days after both intravitreal and systemic administration. Furthermore, hydroxyl‐terminated generation 4.0 PAMAM dendrimers (D4‐OH) show immense promise as ocular nanocarriers to target retinal cells.^[^ ^101^ ^]^ Surprisingly, the nearly neutral charge and tiny size of triamcinolone acetonide‐conjugated D4‐OH could target reactive ma/mi and hypertrophic RPE in a subretinal lipid‐injected CNV rat model without targeting ligands, enabling unrestricted retinal penetration and efficient phagocytosis by ma/mi and RPE (Figure 6b). In another study, Zhou et al. innovated a boronic acid‐rich dendrimer (BARD) using generation 5 PAMAM dendrimers. The BARD was designed for targeted delivery of intracellular superoxide dismutase (SOD) to the ocular posterior segment to reduce acute retinal oxidative stress.^[^ ^121^ ^]^ The study demonstrated that their novel BARD‐mediated SOD nanoformulation effectively reduced cell apoptosis and protected retinal function. The PAMAM dendrimer‐based nanoformulation achieved high cellular uptake without causing immunogenicity or cytotoxicity. These findings imply that the nanoformulation is a highly effective method for intracellularly delivering native proteins and peptides while also preserving their biological activities. Moreover, the dendrimer‐based PAMAM system offers considerable flexibility in precisely regulating the delivery of various biomedical agents through interacting with functional hydroxyl, amino, and/or carboxyl groups on the dendrimers’ terminal surfaces. With the incorporation of a cyclic RGD hexapeptide, this system has been modified to achieve noninvasive, targeted permeation into the posterior segment of the eye.^[^ ^122^ ^]^ Furthermore, the BARD exhibited a highly effective and reliable protein delivery method. Attributing to the presence of an electron‐deficient group, phenylboronic acid (PBA), it was allowed to form binds with the positively charged groups of proteins via combinations of cation‐π and ionic interactions and nitrogen‐boronate complexation. In addition, the positively charged dendrimer interacted with the negatively charged groups of proteins, resulting in a synergistic effect that explains the exceptional delivery effectiveness.^[^ ^123^ ^]^

Nonetheless, dendrimers also have some underlying disadvantages, such as potential toxicities, inadequate assessments of quality control in vivo, sophisticated multistep synthesis, and high preparation costs. These factors indeed impede the development and translation of dendrimers from laboratory research to clinical applications.

#### 3.1.4 Nanogels

Nanogels are cross‐linked three‐dimensional networks of hydrophilic polymers characterized by their tiny nanoscale sizes, exhibiting remarkable characteristics, such as easy preparation and modification, high drug loading capacity, and stable controlled release.^[^ ^124^ ^]^

Recently, for rational drug combination and sustained retinal drug delivery, Gao et al. have exploited an innovative and well‐designed shear‐thinning injectable nanogel by simply mixing betamethasone phosphate (BetP), a commonly used anti‐inflammatory drug in clinical settings, anti‐VEGF, the gold‐standard drug for AMD treatment, with CaCl_2_, totally named BetP‐Gel (Figure 6c).^[^ ^102^ ^]^ Upon IVT, BetP‐Gel provides sustained release of anti‐VEGF, effectively inhibiting vascular proliferation in the retina and attenuating CNV. Additionally, the BetP‐Gel exhibits antioxidant properties, reducing local inflammation. Remarkably, the BetP‐Gel significantly extends the effective treatment duration of conventional anti‐VEGF therapy. As this supramolecular hydrogel consists of clinically approved agents, it holds the potential for translation into clinical use as an AMD treatment, potentially replacing current anti‐VEGF therapies.

### 3.2 Lipid‐Based Nanocarriers

Lipid‐based nanocarriers have garnered extensive attention in medical research due to their capabilities to transport both hydrophobic and hydrophilic compounds, as well as excellent biocompatibility and biodegradability, leveraging their unique attributes such as amphiphilic nanostructures for barrier penetration, tailored modifications for cell targeting, versatile cargo capacity, and controlled release for extended treatment duration.^[^ ^46^ ^]^ Lipid‐based nanocarriers mainly comprise liposomes, lipid nanoparticles, and nanoemulsions.^[^ ^23c^ ^]^

#### 3.2.1 Liposomes

Liposomes are constructed from one or more lipid bilayer membranes, encompassing an aqueous interior. These lipid structures primarily consist of phospholipids and cholesterol. Phospholipids create the bilayer structure, while cholesterol serves to stabilize fluid bilayers, thereby minimizing content leakage from liposomes.^[^ ^125^ ^]^ Liposomes are classic and widely applied nanoplatforms acknowledged by their inherent biocompatibility, biodegradation, and drug‐loading capacity of differing polarities, which have effectively facilitated the delivery of diverse drug categories to the retina, including CRISPR‐Cas9 ribonucleoprotein complexes,^[^ ^126^ ^]^ siRNAs,^[^ ^127^ ^]^ plasmids,^[^ ^128^ ^]^ and proteins.^[^ ^129^ ^]^

Initially, liposomes were explored for delivering anti‐VEGF like bevacizumab. In a study, the concentration of bevacizumab injected into rabbit eyes was compared when delivered either in its free form or encapsulated within liposomes.^[^ ^130^ ^]^ Notably, the group receiving liposomal encapsulation exhibited a fivefold higher drug concentration 42 days post‐injection compared to the free form, elucidating that liposomal encapsulation has the potential to enable more sustained drug release, thereby extending the intervals between injections. Subsequent efforts have aimed to broaden the range of drug cargo that can be incorporated into liposomal formulations, such as receptor tyrosine kinase (RTK) like sunitinib, sorafenib, etc.,^[^ ^131^ ^]^ or cytotoxic drugs like doxorubicin.^[^ ^132^ ^]^ In addition to protein drugs targeting VEGF, certain studies have shifted attention to upstream factors in the disease process, including antioxidation and anti‐inflammation. For instance, liposomes encapsulating platinum (Pt) nanozymes to counteract reactive oxygen species (ROS) were recently reported.^[^ ^133^ ^]^

Enhancing bioavailability in ocular posterior segment delivery relies on stabilized formulations, extended retention periods, and targeted attributes. With the development of nanotechnology, modifying the structure of liposomal formulations has emerged as an innovative approach to stabilize protein drugs. For example, applying a chitosan coating to the liposome has the potential to extend retention time due to its mucoadhesive properties.^[^ ^134^ ^]^ A novel sustained‐release DDS was prepared by modifying APRPG (Ala‐Pro‐Arg‐Pro‐Gly), a synthetic short vessel‐homing peptide specifically targeting VEGFR1 in CNV, with liposome. Thus, the APRPG‐modified liposome improved the hydrophobicity of the encapsulated drug triptolide and enhanced its inhibitory effects on laser‐induced CNV.^[^ ^135^ ^]^ Likewise, triphenylphosphonium (TPP)‐modified liposomes, where TPP is a lipophilic positively charged cation exhibiting selective accumulation within negatively charged mitochondria, and liposome contributed to the cargo‐loading and biocompatibility, were designed to target mitochondria to attenuate RNV and retinal ischemia‐reperfusion injury.^[^ ^133^, ^136^ ^]^ Multivesicular liposomes, featuring separate internal aqueous compartments, offer enhanced encapsulation efficiency for hydrophilic drugs and mitigate initial drug burst release, and the incorporation of nonconcentric lipid layers can further enhance the stability of proteins or peptides.^[^ ^137^ ^]^ Additionally, liposomes can exhibit targeting characteristics by being linked with peptides or proteins with specific functions, as exemplified by the successful use of homing peptides,^[^ ^132^, ^138^ ^]^ RGD peptides,^[^ ^139^ ^]^ etc. Specifically, Li et al. devised a noninvasive liposome for efficient co‐delivery of ellagic acid and oxygen for ameliorating DR, which can be administered intravenously or by eye drops. By utilizing dual modifications, cell‐penetrating peptide and isoDGR (ligands of αvβ3, overexpressed on RPE during neovascular pathogenesis), the liposome could obviously target the damaged RPE to eliminate ROS and down‐regulating the expression of glial fibrillary acidic protein (GFAP), HIF‐1α, VEGF, and p‐VEGFR2 in a DR mouse model.^[^ ^140^ ^]^

Besides, compound liposomes hold great potential for treating FNDs. Lai et al. demonstrated the use of third‐generation PAMAM‐coated compound liposomes for ocular posterior segment delivery.^[^ ^123^ ^]^ In this study, berberine hydrochloride (BBH), an antioxidant, anti‐inflammatory ingredient capable of inhibiting vascular smooth muscle cell proliferation, and Chrysophanol (CHR), an anthraquinone that can inhibit NF‐κB/Caspase‐1 signaling, were exploited as the model drug for an innovative ocular DDS composed of liposomes and PAMAM dendrimers. The coated liposomes demonstrated noticeable protective effects in both human RPE cells and rat retinas following light‐induced oxidative retinal injury, owing to PAMAM increasing the bioavailability and enhancing the capsulation efficiency of agents. Similarly, Li et al. innovated a novel injectable hydrogel loaded with liposomes (cSA@Lip‐HAC) for the treatment of CNV.^[^ ^141^ ^]^ The co‐loading of multitarget angiogenic inhibitor (sunitinib) and hypoxia‐inducible factor inhibitor (acriflavine) was achieved by the liposome, the retention time of which was further extended at the target site by the hydrogel after sub‐tenon's injection, where the liposome and hydrogel achieved to complement each other and together contributing to the biocompatibility, sustained‐release property, and remarkable anti‐CNV property. Overall, these compound systems combining the merits of the liposome and other DDSs showed enhanced anti‐angiogenic effects, thus providing new alternatives for the treatment of neovascular ocular disease.

#### 3.2.2 Lipid Nanoparticles

Lipid nanoparticles can be further categorized into two types: solid lipid nanoparticles (SLNs) and nanostructured lipid carriers (NLCs). SLNs are crafted from lipids exhibiting solidity at either body or room temperature. Typically, they are characterized by low toxicity, favorable biocompatibility, and biodegradability with diameters spanning from 10 to 1000 nm.^[^ ^142^ ^]^ These nanoparticles adopt spherical structures featuring a solid lipid core matrix stabilized by surfactants.^[^ ^143^ ^]^ The core matrix has the capacity to solubilize lipophilic substances. A key advantage of SLNs lies in their notably restricted drug mobility, which extends the duration of drug release. SLNs have the potential to transport payloads like plasmids^[^ ^144^ ^]^ as well as drugs such as tobramycin,^[^ ^145^ ^]^ sunitinib,^[^ ^146^ ^]^ or myriocin^[^ ^147^ ^]^ to the retina. NLCs represent the second generation of SLNs, developed to address certain limitations of SLNs. Comprising a blend of solid and liquid lipids, NLCs exhibit a less structured crystalline arrangement than SLNs, leading to enhanced drug‐loading capacity due to increased available space.^[^ ^148^ ^]^ This disordered structure reduces drug leakage, rendering improved stability and reduced expulsion potential during storage. Unlike SLNs, which may experience drug expulsion due to their highly ordered crystalline structure, NLCs demonstrate enhanced stability and minimized drug leakage.^[^ ^149^ ^]^ These advantages position NLCs as a viable option for efficient retinal drug delivery. NLCs have demonstrated successful delivery of triamcinolone acetonide and palmitoylethanolamide (PEA) to the retina.^[^ ^150^ ^]^

**Figure 7:**
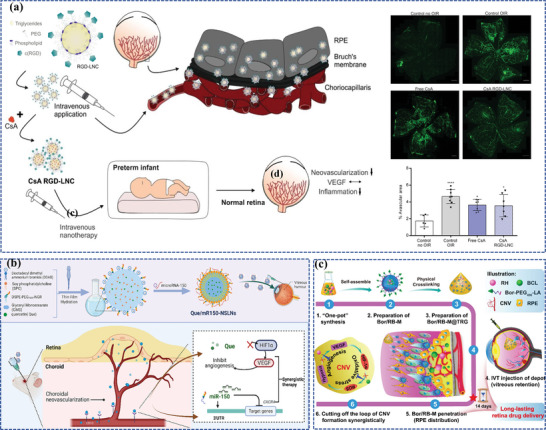
Lipid‐based nanocarriers for ocular posterior drug delivery. a) Schematic illustration of the lipid nanocapsule and therapeutic concept to prevent ROP. Reproduced under the terms of the CC‐BY‐NC.^[^ ^151^ ^]^ Copyright 2022, American Association for the Advancement of Science. b) Schematic illustration of the synthesis and co‐delivery of microRNA‐150 and quercetin of Que/mR150‐NSLNs for treating CNV. Reproduced with permission.^[^ ^142^ ^]^ Copyright 2023, Elsevier. c) Schematic illustration of the synthesis and sustained retinal drug delivery of Bor/RB‐M@TRG, which is capable of sustainedly releasing Bor/RB‐M to the RPE, thereby collaboratively inhibiting the formation of CNV driven by the “angiogenesis‐oxidative stress” loop. Reproduced under the terms of the CC‐BY.^[^ ^153^ ^]^ Copyright 2023, The Authors, published by Springer Nature.

One of the representative examples of lipid nanoparticles (Que/mR150‐NSLNs) was developed by Li et al. for AMD therapy (**Figure** **7**b).^[^ ^142^ ^]^ In this study, researchers formulated SLNs carrying a combination of quercetin (Que) and microRNA‐150 (mR150) while concurrently modifying these particles with an asparagine‐glycine‐arginine (NGR) peptide that exhibits a specific affinity for CD13 receptors, which experience heightened expression on endothelial cells during irregular angiogenesis, thereby enhanced cellular uptake of both drugs and prolonged their therapeutic effects in a mouse model of CNV. Another innovative lipid nanoparticle was a lipid nanocapsule loaded with cyclosporin A (CsA), an FDA‐approved drug that can suppress the VEGF signaling pathway at various intracellular sites, to prevent ROP designed by Bohley et al.(Figure 7a).^[^ ^151^ ^]^ The lipid nanocapsule comprises a core of triglycerides and a phospholipid shell, which enables choroidal extravasation and facilitates particle uptake by interacting with lipoprotein receptors, including the scavenger receptor CD36 on RPE cells, then was outfitted with cyclo‐RGD tethered to the particle corona, contributing to enhancing the specificity and avidity for the target cells and further enabling the nanocapsule to traverse the choroidal endothelial cell barrier. Surprisingly, by a single intravenous injection, the CsA‐loaded nanocapsule not only fulfilled to prevent ROP but also fought against immune system activation and retinal inflammation in a DR mouse model in the following study of the same research team.^[^ ^152^ ^]^

#### 3.2.3 Nanoemulsions

Nanoemulsions are thermodynamically stable, transparent, isotropic, or translucent dispersive systems with nanoscale sizes ranging from 1 to 100 nm, prepared spontaneously by oil, water, surfactants, and co‐surfactants.^[^ ^154^ ^]^ Generally, nanoemulsions can be subdivided into three categories: oil‐in‐water nanoemulsion (O/W), water‐in‐oil nanoemulsion (W/O), and bicontinuous nanoemulsion (B.C).^[^ ^155^ ^]^

Intriguingly, a stable nanoemulsion, referred to as gel‐in‐water (G/W) nanoemulsion, was formulated using beeswax dissolved in castor oil as the inner gel phase of the emulsion, phosphate‐buffered saline (PBS; pH 7.4) as the aqueous phase, and polyoxyethylene hydrogenated castor oil‐60 (HCO‐60) as the emulsifying agent. It effectively delivers hydrophobic drugs to the fundus, which allows for a single administration, eliminating the need for repeated injections that can cause tissue damage and enhancing patient compliance.^[^ ^156^ ^]^ Another example is a composite nanoemulsion for the treatment of AMD, including the combination of lutein (an antioxidant agent), stearyl penetration (a cell‐penetrating agent), a nanoemulsion (a water‐soluble enhancer), and gellan gum (an in situ gelling agent), which can safeguard retinal cells against the damage resulting from hydrogen peroxide (H_2_O_2_) and scavenge intracellular ROS.^[^ ^157^ ^]^ Furthermore, for rational drug combination and sustained retinal drug delivery, Su et al. have successfully created Bor/RB‐M@TRG, an intravitreal‐injectable nanoemulsion depot designed for efficient permeation into the posterior ocular segment and prolonged distribution in the RPE layer for at least 14 days (Figure 7c).^[^ ^153^ ^]^ Bor/RB‐M@TRG is composed of borneol‐decorated rhein and baicalein‐coloaded microemulsions (Bor/RB‐M) as the therapy entity, along with a temperature‐responsive hydrogel matrix serving as the intravitreal depot. Remarkably, a single dose of IVT exhibited potent suppression of the CNV in the wet AMD mouse model.

### 3.3 Inorganic Nanoparticles

Over the past few years, various inorganic nanoparticles, including metallic nanoparticles, carbon dots (CDs), and silica nanoparticles, have been exploited for their potential in drug delivery, gene delivery, and therapeutic applications.^[^ ^24^, ^158^ ^]^ A significant proportion of inorganic nanoparticles, mostly metallic nanoparticles, and CDs, can be considered and developed as nanozymes, which are characterized by intrinsic enzyme‐like characteristics, including peroxidase‐, oxidase‐, haloperoxidase‐, horseradish peroxidase (HRP)‐, catalase (CAT)‐, SOD‐, or glutathione peroxidase (GPX)‐like catalytic activities, which have shown prospects in biomedical applications, owing to their distinct physicochemical properties, including uniform nanoparticle size distribution, significant specific surface area, and antioxidant properties.^[^ ^159^ ^]^ Metallic nanoparticles and CDs also exhibit magnetic, thermal, and light‐responsive characteristics, which have huge potential to develop various smart nanocarriers for the treatment of FNDs (which are discussed more thoroughly in Section 4).^[^ ^158^, ^160^ ^]^ Besides, the physical and chemical characteristics of silica nanoparticles, including their adjustable particle size, substantial specific surface area, and porosity, exceptional biocompatibility, and biodegradability, position them as remarkable nanoplatforms for drug delivery and therapeutic applications emerging in ocular application.^[^ ^161^ ^]^

#### 3.3.1 Metallic Nanoparticles

Metallic nanoparticles hold particular advantages in biomedical engineering attributed to the precise control over their charge, shape, size, and surface modification,^[^ ^162^ ^]^ which are emerging as rising stars in developing ocular nanomedicines.^[^ ^163^ ^]^ Compared to non‐metallic nanoparticles of comparable sizes, the higher density of metallic nanoparticles facilitates more efficient cellular uptake, offering a distinct advantage in the context of ocular posterior drug delivery strategies.^[^ ^164^ ^]^ In the following, we mainly overview metallic nanoparticles that were reported for FND management, including Fe‐, Pt‐, Au‐, and ceria‐based nanoparticles.

#### Fe‐Based Nanoparticles

Among various metallic nanoparticles, Fe‐based nanoparticles, where Fe‐based single‐atom nanozymes (Fe‐N‐C) structures were among the earliest inorganic nanomaterials and one of the most successful translational drugs.^[^ ^165^ ^]^ They exhibit functionally remarkable biocompatibility, electromagnetic functional physical properties, highly catalytic activity, and modifiable biochemical activity in vivo and in vitro.^[^ ^166^ ^]^

**Figure 8:**
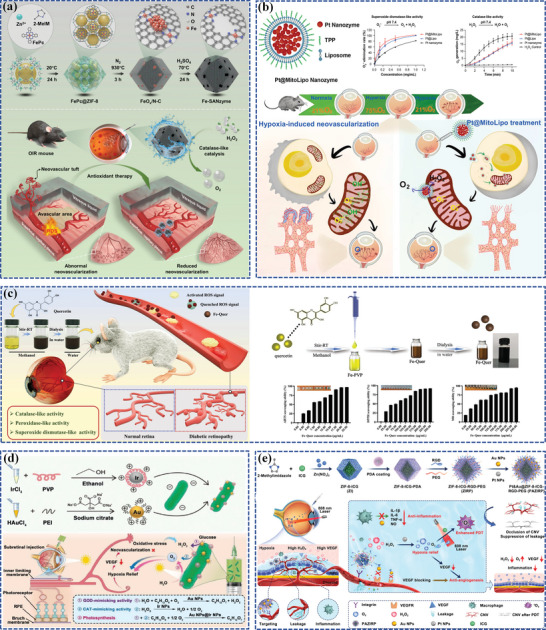
Fe, Pt, and Au‐based nanoparticles as components with enzyme‐like catalysis to develop nanozymes. a) Schematic illustration of Fe‐SANzyme with boosted CAT‐like performance for anti‐angiogenic properties. Reproduced with permission.^[^ ^166b^ ^]^ Copyright 2022, Wiley‐VCH. b) Schematic illustration of Pt@MitoLipo and its anti‐oxidative and anti‐angiogenic properties. Reproduced with permission.^[^ ^133^ ^]^ Copyright 2023, Elsevier. c) Schematic illustration of the preparation of Fe‐Quer NZ and its enzyme‐like activities to relieve pathological neovascularization in DR. Reproduced with permission.^[^ ^166d^ ^]^ Copyright 2023, Wiley‐VCH. d) Schematic illustration of the synthesis process of Cyano@Au@Ir and its therapeutic mechanism against DR through continuous oxygen supply and nanozyme cascade reaction. Reproduced with permission.^[^ ^167^ ^]^ Copyright 2023, Wiley‐VCH. e) Schematic illustration of multifunctional enzymes‐engineering metal‐organic framework nanoplatform (PAZIRP) for enhanced CNV therapy. Reproduced with permission.^[^ ^168^ ^]^ Copyright 2023, Wiley‐VCH.

Recently, Zhang et al. precisely designed a defective Fe‐N4 single‐atom nanozyme (Fe‐SANzyme) exhibiting well‐defined edge‐hosted single‐atom sites, intensified active‐site availability, and abundant mesopores (**Figure** **8**a).^[^ ^166b^ ^]^ The mesoporous structure‐induced formation of edge‐hosted Fe‐N4 moiety of Fe‐SANzyme exhibited a significantly more favorable energy profile thermodynamically compared to the intact atomic configuration. Consequently, it displayed enhanced catalytic performance with higher activity, possessing an ultra‐high affinity for the substrate H_2_O_2_, with a catalytic kinetic KM value (18.80 mm) and a high CAT‐like activity (52.64 U mg^−1^) even with a low iron loading of 0.45 wt%, superior to that of natural CAT and most reported nanozymes. Fe‐SANzyme exhibited exceptional antioxidant properties in endothelial cells. Moreover, as a proof of concept, pathological vascular dysfunction was effectively relieved in two types of animal models, the oxygen‐induced retinopathy (OIR) mouse model and the laser‐induced CNV mouse model, while vascular normalization remained unaffected after IVT of Fe‐SANzyme. The study proposed an innovative strategy for optimizing the catalytic capacities of nanozymes and established the synergistic structure‐activity relationship between single‐atom sites and neighboring defects in CAT‐like catalysis.

However, nanozyme may not completely employ anti‐ROS function with just a single enzyme activity (CAT). Therefore, in order to develop Fe‐based nanozyme with more catalytic activities and further into a feasible DDS, Gui et al. presented the successful synthesis of ultrasmall nanozyme (Fe‐Quer NZ) by combining quercetin, a natural small‐molecule organic compound that has remarkable ROS scavenging capacity but low stability and poor bioavailability in physiological environments, with low‐toxic iron ions by simply introducing a solution of poly(vinylpyrrolidone) (PVP) mixed with iron ions dropwise to enhance the growth of Fe‐Quer NZs followed by ultrafiltered in a sterile water environment (Figure 8c).^[^ ^166d^ ^]^ The nomenclature of Fe‐Quer NZs highlights its excellent mimicry of the enzymatic activities in organisms, including peroxidase, CAT, and SOD, which are necessary for scavenging excess ROS and restoring optimal oxidative stress levels. In vivo assays demonstrated that Fe‐Quer NZs exhibited protective effects against inflammation and oxidative stress‐induced injuries of DR, as well as anti‐angiogenic properties, even at low oral doses. Likewise, to alleviate DR, Gui et al. presented a novel ultrasmall nanozyme (Fe‐DMY NCP), achieved by coupling dihydromyricetin (DMY), which is a naturally occurring flavonoid exhibiting anti‐inflammatory, antioxidant, and anti‐angiogenic activities, with iron ions by a similar method.^[^ ^169^ ^]^ Through mechanistic validation, Fe‐DMY NCPs have the capability to inhibit the Poldip2‐Nox4‐H_2_O_2_ signaling pathway and down‐regulate crucial vascular function indicators. Significantly, these studies highlight the potential of artificial nanoparticles composed of molecules of natural products and metal ions as innovative agents that would provide new insights into catalytic nanomedicine for the treatment of DR and other ROS‐related disorders.

Besides, Prussian blue, a coordination compound, also known as ferric ferrocyanide, also has the potential to develop nanozymes.^[^ ^170^ ^]^ Specifically, considering the ferrous overload and oxidative stress in RPE pathologies associated with AMD,^[^ ^171^ ^]^ a biocompatible Ca^2+^‐substituted iron‐binding Prussian blue nanoparticle KCa[FeIII(CN)^6^] (CaPB) was rationally designed to eliminate labile intracellular ferrous ions.^[^ ^172^ ^]^ CaPB possessed the ability to replace calcium ions within its lattice with ferrous ions due to its high solubility product constant (≈ 3 × 10^−41^). This process caused a dose‐dependent increase in absorption within the range of 500–1000 nm in the ultraviolet spectrum while reducing the characteristic absorption peak of CaPB at 400 nm, indicating the successful exchange between CaPB and ferrous ions. In mice, a single IVT of CaPB demonstrated remarkable efficacy in preventing RPE degeneration and subsequent photoreceptor cell degeneration.

#### Pt‐Based Nanoparticles

Platinum nanoparticles (Pt NPs) also emerged as nanozymes, which have been indicated to have the capacity to exert the antioxidant effect to safeguard cells against oxidative damage.^[^ ^173^ ^]^ The degeneration of photoreceptors in AMD is linked to oxidative stress arising from the high‐intensity aerobic metabolism of rods and cones in the retina, which, if not counterbalanced by endogenous antioxidant mechanisms, can accelerate photoreceptor degeneration.^[^ ^174^ ^]^ Given that, Su et al. utilized a light‐induced retinal degeneration mouse model and discovered that Pt NPs could effectively enhance retinal self‐repair by boosting retinal antioxidant stress response, which demonstrated the potential application value of Pt NPs for treating AMD and other retinal degenerative diseases.^[^ ^175^ ^]^ Furthermore, Cupini et al. assessed the effectiveness of intravitreally injected Pt NPs in either preventing or alleviating light‐induced damage in dark‐reared rats and found that both preventive and post‐lesional IVT of Pt NPs led to increased photoreceptors after light damage, further indicating that Pt NPs can effectively disrupt the detrimental cycle that connects oxidative stress, degeneration, and inflammation by acting as both antioxidants and anti‐inflammatory agents.^[^ ^176^ ^]^ Intriguingly, for RNV therapies, Xue et al. devised a Pt‐based nanozyme functioned with mitochondria‐targeted ability that can eliminate oxidative damage in RNV (Pt@MitoLipo) (Figure 8b).^[^ ^133^ ^]^ In this research, liposomes were used to encapsulate ultrasmall (6–8 nm) Pt NPs to enhance their biocompatibility and tissue permeability and favor Pt NPs to traverse the multi‐layered fundus barriers to reach the retina. Mitochondria‐targeting ability was endowed to the liposomes via TPP modification. It has been verified in vitro and in vivo that Pt@MitoLipo nanozyme was capable of penetrating deep into the fundus, overcoming the intracellular lysosomal barrier, accumulating in mitochondria, and then serving as a SOD/CAT cascade nanozyme under the neutral physiological microenvironment of mitochondria to scavenge ROS and alleviate hypoxia for RNV therapy.

#### Au‐Based Nanoparticles

Gold nanoparticles (Au NPs) are increasingly utilized for biomedical applications, particularly for drug delivery, gene delivery, photoacoustic imaging, and photothermal therapy (PTT).^[^ ^24^, ^177^ ^]^ Depending on the experimental conditions during their formulations, the nanoscale diameters of Au NPs can range from 1 to more than 120 nm. Various shapes can be achieved, including core‐shell nanoparticles,^[^ ^178^ ^]^ nanocages,^[^ ^179^ ^]^ or nanorods where the aspect ratios (length divided by width) can modify their optical properties.^[^ ^180^ ^]^ Au NPs emerged as dependable nanoplatforms for both diagnosis and treatment in the field of ophthalmology.^[^ ^24b^ ^]^

First, Au NPs can function as remarkable contrast agents in photoacoustic imaging and real‐time monitoring of fundus neovascularizations. Intriguingly, Nguyen et al. have developed a series of Au NP‐based nanoplatforms to enhance multimodal photoacoustic microscopy and optical coherence tomography (OCT) molecular imaging of CNV.^[^ ^177^, ^181^ ^]^ A representative example is chain‐like gold nanoparticles (CGNP) clusters combined with RGD.^[^ ^181a^ ^]^ RGD peptides were conjugated with ultrapure CGNP clusters, resulting in a peak wavelength redshift of 650 nm. The formulated nanoparticles demonstrated exceptional biocompatibility and photostability and could biodegrade to allow for efficient elimination from the body. Notably, administering CGNP clusters‐RGD through intravenous injection in the marginal ear vein bound to CNV led to a remarkable seventeen‐fold increase in the signal of photoacoustic microscopy and a 176% surge in the OCT signal. This enhancement is advantageous to visualize and monitor neovascularizations in the subretinal space. Furthermore, given that commonly available Au NPs tend to accumulate in the spleen and liver, which raises concerns regarding their long‐term biological safety, the origin research team upgraded the CGNP clusters further into renally clearable ultraminiature CGNP clusters (small GNCs).^[^ ^177d^ ^]^ Upon disassembly, small GNCs revert to Au NPs with a size smaller than the renal glomerular filtration size cutoff, enabling them to be excreted in urine.

Second, Au NPs can serve as antioxidant agents with glucose oxidase (GOD)‐like activity, as reported before.^[^ ^182^ ^]^ Taking advantage of that property, Zhou et al. further designed an exceptional cascaded nanozyme combining Au NP as a part of it (Figure 8d).^[^ ^167^ ^]^ In this study, cyanobacteria were adopted to load Au NPs exhibiting GOD‐like activity and iridium nanoparticles (Ir NPs) exhibiting CAT‐like activity, forming Cyano@Au@Ir double nanozymes cascade system. The Au NPs initially degraded glucose into H_2_O_2_, which was further converted into H_2_O and O_2_ by the Ir NPs, completing the cascaded hypoglycemic reaction. Taking advantage of the unique light transmittance of the eyeball and light accumulation in the retinal area, cyanobacteria continuously produce O_2_, alleviating the hypoxia in the microenvironment, leading to reduced expression of VEGF and hypoxia‐inducible factors. Additionally, the high levels of peroxide in the DR microenvironment are efficiently eliminated by Ir NPs, exhibiting an anti‐inflammatory property. This innovative treatment approach not only degrades blood glucose but also provides continuous O_2_ supply and scavenges free radicals, achieving comprehensive microenvironment regulation and offering potential solutions for addressing fundus complications in DR. Intriguingly, for more precise PDT and microenvironment amelioration, Jin et al. designed a novel nanoplatform by decorating zeolitic imidazolate framework‐8 (ZIF‐8) with Pt NPs and Au NPs to load indocyanine green (ICG) (Figure 8e).^[^ ^168^ ^]^ The nanoplatform can penetrate the ILM through Müller cells’ endocytosis and target CNV by surface‐grafted RGDs after IVT. The Pt NPs act as CAT‐like enzymes to catalyze excessive H_2_O_2_ in the CNV microenvironment, relieving hypoxia and enhancing PDT occlusion of neovascular, and the Au NPs exhibit significant anti‐inflammatory and anti‐angiogenic properties by regulating macrophages and blocking VEGF. Compared with verteporfin treatment, the nanoplatform group shows a 90.2% downregulation in HIF‐1α mRNA expression and an 81.7% downregulation in VEGF mRNA expression. The nanoplatform achieves comprehensive CNV treatment based on high drug‐loading capacity and biosafety.

Third, Au NPs exhibit high photothermal conversion efficiency, primarily due to their remarkable localized surface plasmon resonance properties. An example was for the purpose of controlled delivery of bevacizumab, which consists of Au NPs embedded within agarose gels, and it was specifically designed for the treatment of AMD.^[^ ^183^ ^]^ The incorporation of Au NPs in this system enables photothermal conversion occurring in an on/off cycle controlled by light irradiation, resulting in a localized temperature increase within the hydrogel, inducing a sol‐gel transition, thus promoting drug diffusion into the surrounding environment. In the same context, Au NPs were incorporated into dual‐responsive liposomes (temperature‐/pH‐responsive), which allows for selective activation within the acidic compartments of cells, such as endosomes and lysosomes, when stimulated by visible and near‐infrared (NIR) light.^[^ ^184^ ^]^

#### Ceria‐Based Nanoparticles

**Figure 9:**
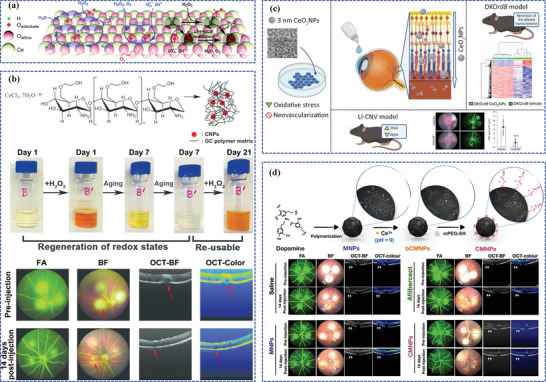
Ceria nanoparticles as components with enzyme‐like catalysis to develop nanozymes. a) Schematic illustration of the antioxidant capacities of nanoceria scavenging superoxide and H_2_O_2_. Reproduced with permission.^[^ ^186^ ^]^ Copyright 2019, Royal Society of Chemistry. b) Schematic illustration of GCCNP synthesis and its auto‐regenerative antioxidant significantly attenuates CNV. Reproduced with permission.^[^ ^192^ ^]^ Copyright 2017, American Chemical Society. c) Schematic illustration of repeated topical administration of 3 nm CeO_2_NPs eyedrop reverted disease atrophic phenotype and reduced CNV lesions in mouse models. Reproduced with permission.^[^ ^195^ ^]^ Copyright 2023, American Chemical Society. d) Schematic illustration of the preparation of MNPs and CMNPs, and CMNPs outperformed aflibercept as well as MNPs and effectively alleviated CNV with just a single‐dose administration. Reproduced with permission.^[^ ^196^ ^]^ Copyright 2023, Elsevier.

Ceria‐based nanoparticle, also called nanoceria, is a type of nanozyme possessing remarkable auto‐regenerative antioxidant properties owing to the reversible conversion of Ce^3+^ ions to Ce^4+^ ions in ceria nanoparticle (**Figure** **9**a).^[^ ^24^, ^185^ ^]^ This unique characteristic enables nanoceria to effectively eliminate harmful ROS, having multi‐enzyme mimetic capacities of CAT, SOD, oxidase, and phosphatase, making it a promising candidate for managing oxidative stress‐related diseases, particularly FNDs.^[^ ^186^ ^]^ Additionally, nanoceria can function as a versatile nanocarrier for delivering agents to ocular tissues, further expanding its potential applications in the field.^[^ ^187^ ^]^

A pioneering study demonstrated that nanoceria effectively mitigated retinal degradation induced by light damage and rescued visual function by scavenging cell peroxides.^[^ ^188^ ^]^ Subsequent studies have provided further evidence of the long‐term neuroprotective properties of nanoceria by modulating microglial activation and migration within the subretinal space, which serves an indispensable role in the immune response during cellular damage or inflammatory microenvironments.^[^ ^189^ ^]^ Localization of FITC‐labeled nanoceria within the cytoplasm of RPE cells has been observed, as well as has demonstrated the ability to modulate autophagy, a cellular process involved in maintaining cell homeostasis, to effectively prevent degeneration and subsequent death of RPE cells.^[^ ^190^ ^]^ Furthermore, it was demonstrated that the IVT of nanoceria following bright light damage effectively decreased the buildup of autofluorescent deposits, which are considered a hallmark of retinal stress in the progression of AMD.^[^ ^190^ ^]^ Nanoceria also demonstrated inhibition of pro‐angiogenic growth factors and pro‐inflammatory cytokines in genetic‐induced AMD models, including very‐low‐density lipoprotein receptor (Vldlr) knock‐out mice, which could potentially lead to CNV.^[^ ^191^ ^]^ However, despite these promising findings, there is still a need to establish optimal antioxidant formulations, as traditional nanoceria formulations have certain drawbacks, such as limited solubility and relatively low efficiency. Recent research indicated that glycol chitosan coating by ammonium hydroxide precipitation approach could remarkably enhance the stability and solubility of nanoceria (Figure 9b).^[^ ^192^ ^]^ Glycol chitosan‐coated ceria nanoparticles (GCCNPs) demonstrated the suppression of H_2_O_2_‐induced endothelial cell migration and tube formation in vitro. Moreover, a single intravitreal dose of GCCNPs scavenged intracellular ROS and subsequently downregulated VEGF expression. This ultimately resulted in fewer CNV lesions, counteracting the progression of a laser‐induced CNV mouse model. Furthermore, Wang et al. demonstrated that the combination of GCCNP with an alginate–gelatin‐based injectable hydrogel reduced AMD‐like atrophy and rapidly recovered photoreceptor and RPE cells within five weeks, while GCCNP alone would take up to 10 weeks, which is attributed to the synergistic antioxidant capacities of GCCNPs and the hydrogel.^[^ ^193^ ^]^ The same team further devised oligo‐chitosan‐coated ceria nanoparticles (OCCNPs) alginate‐loaded hydrogels with sustained release, which maintained the cell viability of ARPE‐19 (adult retinal pigment epithelial cell line) cells in H_2_O_2_ and lipopolysaccharides (LPS)‐induced in vitro environment.^[^ ^194^ ^]^ Intriguingly, in order to achieve ocular topical delivery of nanoceria to treat AMD, Badia et al. prepared a formulation that contains small (3 nm), non‐aggregated nanoceria particles (CeO_2_NPs) (Figure 9c).^[^ ^195^ ^]^ In this study, the synthesis process began with sodium citrate‐complexed cerium ions instead of the traditional cerium nitrate and utilizing tetramethylammonium hydroxide (TMAOH) as the base, resulting in the stabilization of single 3 nm CeO_2_NPs in water. It is noteworthy that oxygen vacancies tend to increase as the particle size decreases due to the increased surface‐to‐volume ratio, contributing to the CeO_2_NPs reaching the retina following topical administration. Significantly, after the CeO2NPs treatment, the altered retinal transcriptome was reversed in the retinal degenerative mouse model DKOrd8 towards that of healthy control mice, accompanied by signs of reduced inflammation and the arrest of degeneration.

Specifically, to enhance MNPs, mentioned above in Section 3.1.1,^[^ ^104^ ^]^ as alternative therapeutic effects against AMD, Kwon et al. designed a more innovative antioxidant nanomedicine strategy based on nanoceria‐coated melanin‐PEG nanoparticles (CMNPs) by utilizing the inherent antioxidant capacities of MNPs to autorenew nanoceria (Figure 9d).^[^ ^196^ ^]^ CMNPs outperformed aflibercept as well as MNPs and effectively alleviated oxidant damage in a laser‐induced CNV mouse model with just a single‐dose administration, maintaining its therapeutic effects for up to three months.

#### 3.3.2 Carbon Dots

CDs are 0D nano‐sized particles possessed with great research interest owing to their unique optical characteristics, favorable biocompatibility, low toxicity, and eco‐friendly nature, which make them extensively exploited in biosensing, biological imaging, drug delivery, and photodynamic/photothermal‐related applications.^[^ ^106^, ^197^ ^]^ Based on their properties, such as surface functional groups and carbon core structures, CDs are subdivided into four types: carbon nanodots (CNDs), carbonized polymer dots (CPDs), carbon quantum dots (CQDs), and graphene quantum dots (GQDs).^[^ ^198^ ^]^ CDs can be easily functionalized due to the abundance of surface functional groups, presenting outstanding sensing properties, including specificity, selectivity, and multiplex detectability.^[^ ^158a^ ^]^

**Figure 10:**
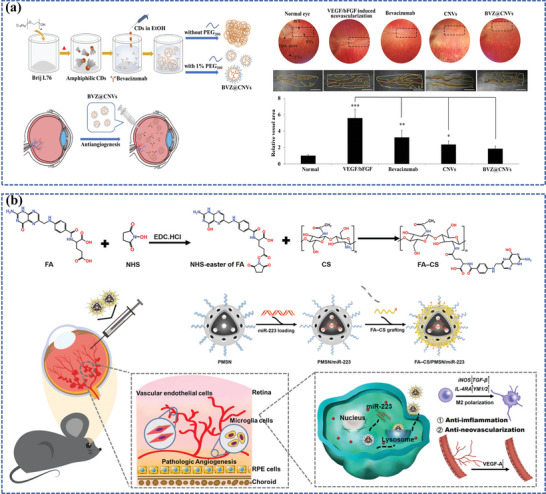
a) Schematic representation of the preparation of carbon nanovesicles from Brij L76 and encapsulation of bevacizumab and its anti‐angiogenic activity in fundus images. Reproduced with permission.^[^ ^203^ ^]^ Copyright 2023, Elsevier. b) Schematic illustration of the preparation and the immunomodulatory function of miRNA‐223‐based MSN nanoplatform for targeted therapy in ROP. Reproduced with permission.^[^ ^204^ ^]^ Copyright 2023, Elsevier.

Currently, CDs and CD‐based nanomaterials have been explored in ophthalmology, including ocular imaging, as well as therapeutic applications for ocular diseases, especially FNDs.^[^ ^158^, ^199^ ^]^ Qu et al. reported a simple approach to synthesizing selenium (Se) and nitrogen (N) co‐doped CDs (Se‐N‐CDs) from low‐cost citric acid, ethane diamine, and selenium powder.^[^ ^200^ ^]^ After doping Se into CDs, Se&N‐CDs extend the absorption range from the ultraviolet region to the visible light region (≈497 nm). Moreover, the Se‐N‐CDs showed a relatively high quantum yield of ≈ 52% in the green emission region, which can be ascribed to the low electronegativity of the newly introduced Se element. Notably, detailed images of the capillary bed and vessels were exhibited after injection in the fundus fluorescein angiography (FFA) images. Characterized by low toxicity, favorable biocompatibility, better penetration, and control over the longer wavelength, it serves as a potential tool for noninvasive imaging and diagnosis. In 2015, the anti‐angiogenic properties of synthesized CQDs were first navigated by Shereema et al.^[^ ^201^ ^]^ The chick chorioallantoic membrane (CAM) treated with CQDs contributed to reduced density of branched vessels and significantly downregulated levels of pro‐angiogenic growth factors as well as hemoglobin, exhibiting that CQDs are capable of inhibiting the process of angiogenesis.^[^ ^201^ ^]^ GQD is reported as a novel biocompatible nanomedicine to participate in the pathological RNV in an OIR mouse model. Compared with the control groups, the number of capillary‐like structures induced in the GQDs‐treated groups. Further cell function experiment reveals that GQDs downregulate the levels of periostin via signal transducer and activator of transcription 3 (STAT3) and subsequently modulated cell cycle‐related protein levels through the extracellular signal‐regulated kinases (ERK) pathway in a dose‐dependent manner, which was confirmed in vivo mouse model experiment, indicating that GQDs could be exploited to treat FNDs by regulating STAT3/periostin/ERK signaling pathway.^[^ ^24c^ ^]^ Besides, CDs and CD‐based nanomaterials were utilized as DDSs loading agents to the desired locations. Shoval et al. reported that CDs functionalized with the anti‐VEGF agents show elevated penetration of the eye structure, and their strong fluorescence is beneficial to noninvasive intraocular concentration monitoring.^[^ ^202^ ^]^ Furthermore, due to the inhibitory functions in the formation of choroidal blood vessels, the anti‐VEGF‐modified CDs are versatile in the application of fundus neovascular diseases, such as AMD and DR. The carbon nanovesicles formed by self‐assembly of CDs (derived from one‐step mild carbonization of Brij L76) were exploited for carrying and continuous release of bevacizumab to inhibit VEGF‐stimulated angiogenesis (**Figure** **10**a).^[^ ^203^ ^]^ The bevacizumab‐loaded carbon nanovesicles (BVZ@CNVs) effectively depress VEGF‐induced endothelial cell proliferation and migration in vitro and relieve the symptoms of angiogenesis induced by VEGF in vivo rabbit model. In comparison to liposomes and polymersomes, BVZ@CNVs possess the advantages of quickness, directness, good biocompatibility, and cost‐effectiveness, which provides a prospective strategy for treating AMD in the future. The above‐mentioned studies present necessary insights to better understand CDs, the versatile therapeutic platform applied in ocular disease diagnosis, ocular imaging, and treatments. A myriad of functionalized CDs and CD‐based nanomaterials are emerging as a novel therapeutic paradigm in the basic study and clinical translation, and more questions, such as mode of administration, pharmacodynamics, pharmacokinetics, and safety issues, remain to be explored.

#### 3.3.3 Silica Nanoparticles

Silica nanoparticles hold strong appeal for various applications owing to their straightforward synthesis, colloidal stability, adjustability of particle size, surface functionalization capabilities, biocompatibility, and the potential for scalable production.^[^ ^161^ ^]^ Significantly, Silica nanoparticles have been demonstrated to bolster the stability of various cargo substances while preserving their specific chemical and physical attributes.^[^ ^205^ ^]^ Mesoporous silica nanoparticles (MSNs), with pore diameters of 2–50 nm, are blessed with unprecedented advantages over other traditional nanocarriers due to their unique physical characteristics (e.g., large pore volume, mesoporous structure, and high surface area). Apart from their significant roles in drug delivery, MSNs serve a potential role in cellular uptake, biosensing, diagnosis, and therapy.^[^ ^206^ ^]^

Recently, Sun et al. synthesized MSN‐encapsulated bevacizumab nanoparticles, which demonstrated prolonged residency of bevacizumab in vitreous or aqueous humor, sustaining drug concentration and no obvious cytotoxicity and tissue toxicity. Furthermore, MSN‐encapsulated bevacizumab nanoparticles elevated the anti‐VEGF effects on RNV in vivo. These results revealed that MSN‐encapsulated bevacizumab could be a promising strategy against FNDs.^[^ ^206c^ ^]^ In addition, Grondek constructed fusogenic lipid‐coated MSNs loaded with VEGF‐A siRNA, which exploited the advantages of excellent biocompatibility and biodegradability of the porous silicon materials, finally decreasing the retinal neovascular leakage.^[^ ^207^ ^]^ In another study, folic acid‐chitosan (FA‐CS)‐modified MSN (PMSN) was carried with microRNA‐223 (miR‐223), a regulator of microglial polarization toward the anti‐inflammation phenotype which helps impress inflammation and subsequently pathological angiogenesis (Figure 10b).^[^ ^204^ ^]^ The delivery system helped target activated microglia, modulated microglial polarization from M1 to M2 state, showed anti‐VEGF activity in cultured microglial cells, and prominently reduced vascular area with downregulated levels of M1 cytokines and upregulated levels of M2 cytokines in the mouse OIR model of ROP. The nanoparticle platform offers a promising therapeutic strategy for FNDs by delivering miR‐223 to modulate microglial polarization in the foreseeable future. Currently, silica is generally recognized as safe and has been approved by the FDA to be exploited for a patient with metastatic melanoma in a clinical trial.^[^ ^208^ ^]^ As a prospective research area of nanomedicine, more researchers need to decipher the underlying effects of MSN encapsulation in ocular disease‐related studies and accelerate the translation from the basic to the clinic.

## 4 Smart Nanocarrier‐Based Nanomedicines for the Treatment of FNDs

To effectively provide drug release that is finely tuned in terms of location, timing, and dosage, the concept of designing nanocarriers tailored to the pathological microenvironment of FND is an appealing proposition. Nevertheless, in the absence of a trigger mechanism, drug release from nanocarriers remains unregulated, potentially causing premature drug release before reaching the intended target site, thus impeding drug bioavailability and compromising overall efficacy.^[^ ^209^ ^]^ To solve this problem, some nanocarriers with “smart” capabilities have emerged as a promising approach to improve the sensitivity and specificity for drug delivery and are rising stars in managing diseases, including FNDs.^[^ ^26^, ^27^, ^210^ ^]^ Smart nanocarriers are based on nanomaterials that respond sharply to small changes in physical or chemical conditions, which include endogenous stimuli, also called pathological microenvironment biomarkers (enzymes, ROS, pH, glucose, adenosine triphosphate (ATP), temperature, ions, hypoxia, nucleic acids, other inflammation signals, etc.) and exogenous stimuli (light, ultrasound, magnetic fields, electric, etc.), with relatively large phase or property changes, thus precisely releasing the desired concentration of active drug at the site of pathology.^[^ ^211^ ^]^ These nanocarriers are also variously referred to as “stimuli‐responsive,” “environmentally‐sensitive,” “microenvironment‐responsive,” “bio/biomarker‐responsive” nanosystems, etc.^[^ ^26^, ^27^, ^211^, ^212^ ^]^

Notably, there are important pathological microenvironmental biomarkers of FNDs (abnormal enzyme expression, ROS overload, and pH changes) and available exogenous stimuli (light and ultrasound), which can be used to develop smart nanocarrier‐based nanomedicines for FND management:

1) Enzymes: Enzymes play a significant role in physiological metabolism. Importantly, abnormal enzyme activities are closely related to the pathologies of various diseases, such as inflammations, cancers, and infections.^[^ ^213^ ^]^ The enzyme‐responsive nanosystem is emerging as a promising approach for achieving precisely targeted and controlled drug delivery.^[^ ^214^ ^]^ Intriguingly, fundus neovascularization lesions are known to be enriched with matrix metalloproteinases (MMPs), making them exploited as the trigger for drug release.^[^ ^215^ ^]^ Besides, some nanoplatforms combined with an enzyme‐responsive element emerged, giving themselves functions for enzyme‐instructed self‐assembly (EISA), which presents a remarkable prospect for realizing targeted and prolonged accumulation of agents at the site of the lesion.^[^ ^216^ ^]^
2) ROS: ROS serve as cell signaling pathway facilitators under normal physiological conditions, contributing to normal bodily functions.^[^ ^217^ ^]^ However, in various ocular pathologies, inflammatory reactions can lead to excessive production of ROS, which is associated with conditions such as AMD,^[^ ^3^, ^38^ ^]^ DR,^[^ ^42^, ^166^ ^]^ ROP,^[^ ^8^, ^204^ ^]^ glaucoma,^[^ ^218^ ^]^ cataract,^[^ ^219^ ^]^ dry eye disease,^[^ ^220^ ^]^ and corneal neovascularization.^[^ ^221^ ^]^ Therefore, ROS can serve as a trigger for release and also facilitate substantial cargo accumulation within these ocular microenvironments.^[^ ^222^ ^]^ ROS group comprises various molecular entities, including H_2_O_2_, singlet oxygen (^1^O_2_), peroxide (O_2_^−^), superoxide (O_2_•^−^), and hydroxyl radical (•OH), among several others.^[^ ^217b^ ^]^
3) pH: The extracellular pH of healthy tissues and blood is maintained at 7.4, whereas the intracellular pH is typically around 7.2.^[^ ^223^ ^]^ Different from healthy tissues, pathological lesions usually exhibit a lower pH than healthy tissues, and the pH gradient in most lesions is inverted (intracellular pH > extracellular pH).^[^ ^211^, ^224^ ^]^ Besides, as widely acknowledged, early endosomes are situated within an acidic environment characterized by a pH range of 6.5 to 5.5. Nanomedicine must overcome endosomal trapping to release the cargo into the cell cytosol. Hence, the pH at different disease sites plays a pivotal role in achieving targeted delivery of therapeutics, and pH‐responsive modifications to nanocarriers become necessary to some degree.
4) Light: The eye stands as the only organ system that enables deep penetration of light into the human body. The exceptional transmittance of the cornea and lens enables specific wavelengths of radiation to pass through the eye noninvasively, reaching the posterior segment.^[^ ^225^ ^]^ Accordingly, the eye stands out as the most advantageous human organ when it comes to the selective utilization of light‐responsive approaches. Due to its ability to be applied remotely with high precision in terms of both spatial and temporal aspects, the light stimulus is particularly intriguing for drug targeting and control of release.^[^ ^84^ ^]^ UV radiation is known to have limited penetration depth in soft tissues, which restricts ultraviolet‐triggered drug delivery to targets that can be directly irradiated, such as the eye. To overcome this limitation, groups sensitive to higher wavelength radiation, such as NIR, have been developed.^[^ ^226^ ^]^
5) Ultrasound: Ultrasound has long been employed for diagnostic applications, particularly in biomedical imaging.^[^ ^227^ ^]^ More recently, ultrasound has been harnessed for therapeutic applications, particularly in the context of drug and gene delivery. Notably, ultrasonic energy has proven effective in triggering the release of drugs from both biodegradable and non‐biodegradable materials, as evidenced by experiments both in vitro and in vivo.^[^ ^228^ ^]^ Control over the permeation of bioactive pharmacological agents into tissues can be achieved by adjusting parameters such as ultrasound frequency, exposure time, and duty cycle.^[^ ^229^ ^]^

Despite numerous research being reported in the field of smart nanocarriers,^[^ ^226^, ^230^ ^]^ to the best of our knowledge, few reviews summarized the advances concerning FNDs. Therefore, we comprehensively overview emerging trends in smart nanocarriers for FND management, anticipating that it will help related researchers to keep up to date with the latest progress and stimulate new thinking about innovative smart nanocarrier‐based nanomedicines for treatment of FNDs.

### 4.1 Enzyme‐Responsive Nanocarrier‐Based Nanomedicines

Concerning FND management, enzyme‐responsive nanocarriers are still in their early beginnings. Enzyme‐triggered drug release involves two key aspects. First, drug release is initiated when the encapsulating component becomes a substrate for specific enzymes, leading to changes in its chemical and physical properties. Second, it can also occur owing to alterations of the internal interactions within the nanocarriers housing the drug payload, causing instability in the nanocarriers and subsequent release of the payload.^[^ ^231^ ^]^

**Figure 11:**
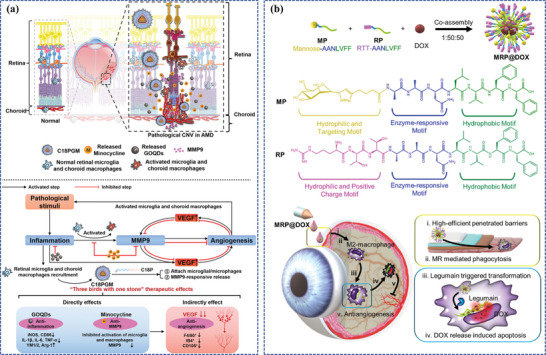
Enzyme‐responsive nanocarrier‐based nanomedicines for the treatment of FNDs. a) Schematic illustration of the preparation of C18PGM as a novel DDS for CNV treatment via counteracting inflammation, suppressing MMP9 activity, and ultimately achieving anti‐angiogenic effects. Reproduced with permission.^[^ ^199^ ^]^ Copyright 2023, Wiley‐VCH. b) Schematic illustration of co‐assemble components of MRP@DOX, which are constructed as GPNTs, and the process to reduce neovascularization. Reproduced with permission.^[^ ^236^ ^]^ Copyright 2022, Elsevier.

The process of neovascular invasion requires the disassembly and remodeling of the extracellular matrix. Within the MMPs family, gelatinases, specifically MMP2 and MMP9, play a crucial role in responding to extracellular matrix regulation.^[^ ^232^ ^]^ Moreover, MMPs can maintain a high expression level through a positive feedback pathway with VEGF,^[^ ^233^ ^]^ making them potentially serve as therapeutic targets for interventions aimed at modulating neovascularization.^[^ ^234^ ^]^ For example, Yahan et al. report a DDS using the FDA‐approved amphiphilic agent triglycerol monostearate to encapsulate verteporfin, which is stimuli‐responsive to the increased expression of MMPs in the CNV lesions.^[^ ^235^ ^]^ Similarly, Tian et al. designed an MMP‐responsive nanomedicine based on exosomes to suppress CNV.^[^ ^215b^ ^]^ Recently, Huang et al. invented an MMP9‐responsive minocycline‐loaded nanomedicine, where the nanocarrier was synthesized by chemically bonding graphene oxide quantum dots (GOQDs) to an octadecyl‐modified peptide (C18P) that can be cleaved by MMP9 specifically. Minocycline, a specific ma/mi inhibitor, was then loaded into the MMP9‐responsive nanocarrier to develop the nanomedicine C18PGM (**Figure** **11**a).^[^ ^199^ ^]^ GOQDs worked as natural antioxidants, exerting anti‐inflammatory capacities, and minocycline could be released responsive to MMP9, thereby suppressing ma/mi activation and MMP9 activity. Utilizing the CNV mouse model, the C18PGM exhibited notable inhibitory activity against MMP9 and demonstrated anti‐inflammatory effects, followed by anti‐angiogenic effects, disrupting the vicious cycle of CNV and achieving a triple therapeutic effect encompassing anti‐inflammatory, anti‐MMP9, and anti‐VEGF actions—“three birds with one stone”. Furthermore, co‐treatment with anti‐VEGFs and C18PGM contributed to optimal efficiency of suppression of CNV, providing a promising paradigm for combinatorial therapy in the treatment of FNDs.

Next, the EISA phenomenon has garnered significant attention in the field of advanced drug delivery, which is a novel form of enzyme‐triggered drug release.^[^ ^216^, ^237^ ^]^ Supramolecular assemblies play crucial roles in living organisms, contributing to the complexity of biological pathways and the compartmentalization of cells. Given the dynamic and crowded cellular environment, diverse strategies can be employed to regulate the formation of DDSs’ assemblies.^[^ ^216a^ ^]^ Therefore, Hu et al. utilized the concept of EISA in the design of a phosphorylated peptide‐drug precursor (IBF‐HYD‐GFFpY) based on ibuprofen.^[^ ^216b^ ^]^ This precursor was designed to undergo self‐assembly in vivo through the catalytic action of alkaline phosphatase (ALP) present in the tear fluid, enabling efficient ocular drug delivery. Importantly, for FND therapy, Li et al. devised co‐assembled glycopeptide nanotransforrs (GPNTs), MRP@DOX, comprising three parts: glycopeptide, cationic peptide, and DOX (Figure 11b).^[^ ^236^ ^]^ The nanosystem demonstrated several advantages: 1) electrostatic interaction between the positively charged MRP@DOX and the negatively charged mucin layer, which prolonged retention time; 2) the mannose ligand of MRP@DOX facilitated phagocytosis by M2 macrophages; 3) an enzyme‐responsive motif cleaved by lysosomes resulted in the self‐assembly of the hydrophobic peptide, forming nanofibers. These nanofibers significantly prolonged the retention time compared to nanoparticles, contributing to a 44.7% DOX retention in cells at 24 h than that of the non‐transformed controls (MAP@DOX: 5.1%). In an OIR mouse model, MRP@DOX significantly reduced the pathological neovascularization and recovered the physiological angiogenesis.

### 4.2 ROS‐Responsive Nanocarrier‐Based Nanomedicines

The underlying release mechanisms of diverse ROS‐responsive nanosystems can be summed up as follows: ROS initially trigger alterations in nanocarrier solubility, leading to nanocarrier degradation or ROS‐induced cleavage of nanocarriers, ultimately culminating in ROS‐induced cleavage of carrier‐drug linkages. Depending on the incorporated payload, ROS‐responsive nanocarriers can encapsulate agents via hydrophobic interactions, covalent bonds, or electrostatic interactions, resulting in distinct patterns of cargo release, which encompass compositions involving thioether, thioketal, selenium, tellurium, polysaccharide, aminoacrylate, boronic ester, peroxalate ester, and polyproline.^[^ ^238^ ^]^

**Figure 12:**
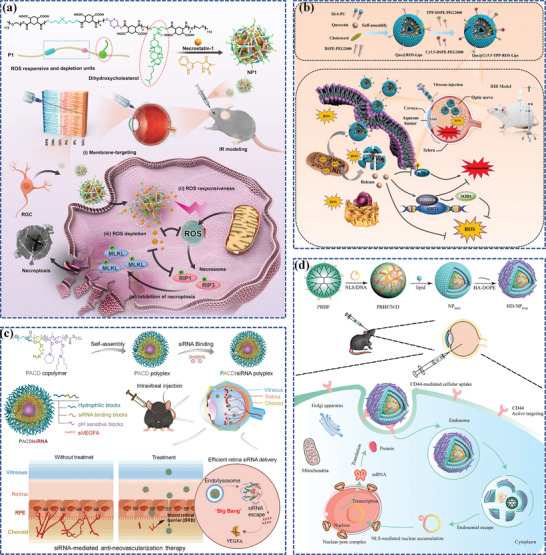
ROS‐responsive and pH‐responsive nanocarrier‐based nanomedicines for the treatment of FNDs. a) Schematic illustration of the design of ROS‐responsive drug carrier polymer targeting the cell membrane, inhibiting necroptosis, and depleting ROS for retinal ischemia‐reperfusion injury. Reproduced with permission.^[^ ^218^ ^]^ Copyright 2022, American Chemical Society. b) Schematic illustration of mitochondria‐targeted ROS‐responsive liposomal quercetin attenuating retinal ischemia‐reperfusion injury via regulating SIRT1/FOXO3A and p38 MAPK signaling pathways. Reproduced under the terms of the CC‐BY.^[^ ^136^ ^]^ Copyright 2022, The Authors, published by Wiley Periodicals LLC. c) Schematic illustration of pH‐responsive polymer PACD promoting cytosolic siRNA release in retinal cells and abrogating hypoxia‐induced RNV. Reproduced with permission.^[^ ^242^ ^]^ Copyright 2024, Elsevier. d) Schematic illustration of a core‐shell delivery nanosystem combining DOPE as the outer shell, lipid bilayer as the inner shell, and dendrimers loaded with nuclear localization signal as the inner core. Reproduced with permission.^[^ ^243^ ^]^ Copyright 2021, Elsevier.

ROS‐responsive nanocarriers appear to be widely used in managing various diseases. While the exploration of ROS‐responsive nanocarriers for ocular applications remains incomplete, some researchers have aimed to unlock the potential of such systems. For instance, Niu et al. used glycol chitosan as a nanocarrier and 4‐carboxyphenylboronic acid pinacol ester as a ROS‐responsive group to encapsulate antifungal drug, voriconazole and then developed a ROS‐controlled release polymeric nanocarrier (GC‐EB) to treat fungal keratitis.^[^ ^239^ ^]^ Unfortunately, the application of ROS‐responsive nanocarriers in FND management is almost a desert. Surprisingly, as an emerged oasis in the desert, Jiang et al. investigated novel PDA nanoparticles loading anti‐angiogenic protein for the treatment of AMD, not only possessing inherent ROS‐scavenging properties but also controllably releasing anti‐angiogenic drugs responsive to ROS upgradation.^[^ ^240^ ^]^ Moreover, several ROS‐responsive nanocarrier‐based nanomedicines for retinal ischemia‐reperfusion injury are also illuminating for FND therapy. A representative example is a ROS‐responsive drug carrier polymer developed by Rong et al. (**Figure** **12**a) that responds to the retinal ischemia‐reperfusion microenvironment, featuring thioketal bonds and a 1,4‐dithiane unit in its main chain, which is ROS‐responsive and helps in reducing ROS via the breakdown of thioketal bonds and oxidation of sulfur ether in the 1,4‐dithiane unit.^[^ ^218^ ^]^ This polymer was adopted to encapsulate an inhibitor of necroptosis, necrostatin‐1, into nanoparticles to scavenge ROS, downgrade the necroptosis pathway, altered inflammatory responses, and oxidative stress both in vitro and in vivo of a retinal ischemia‐reperfusion injury model. Likewise, Zhao et al. designed a mitochondria‐targeted ROS‐responsive liposomal quercetin, Que@TPP‐ROS‐Lips, for attenuating retinal ischemia‐reperfusion injury (Figure 12b).^[^ ^136^ ^]^ In their previous study, ROS‐responsive lipids (Di‐S‐PC) formed from thioether phosphatidylcholines, wherein the conventional fatty acid chain of a lipid is substituted by two tails connected through thioether linkages, were reported.^[^ ^241^ ^]^ This ROS‐responsive feature can be employed in liposomes, enabling the creation of nanocarriers with excellent biocompatibility that target oxidative stress microenvironment.

### 4.3 pH‐Responsive Nanocarrier‐Based Nanomedicines

The pH responsiveness of smart nanocarriers can be attained either through the degradation of acid‐cleavable bonds or the protonation of ionizable groups.^[^ ^231^ ^]^ For instance, chitosan exhibits insolubility under physiological conditions, creating a protective glassy external surface that prevents the leakage of loaded drugs.^[^ ^244^ ^]^ Conversely, when exposed to an acidic environment, chitosan chains extend to a greater extent, thereby enhancing the disintegration and release of agents encapsulated.^[^ ^245^ ^]^ On the other hand, acid‐cleavable bonds include hydrazone, orthoester, imine, vinylether, oxime, acetals, etc., which can be cleaved by acid surrounding medium, thus disrupting the amphiphilic balance of the copolymers and triggering cargo release.^[^ ^246^ ^]^ According to where drug release is triggered, pH‐responsive nanocarriers can be categorized as either extracellular pH‐responsive or cytosolic pH‐responsive nanocarriers.^[^ ^211^ ^]^ Extracellular pH‐responsive nanocarriers can release the drug in the pathological microenvironment, responsive to the extracellular pH, before they are taken up by cells. Although these polymers are more commonly employed in the ocular anterior disease treatment, they are not extensively applied in the management of FNDs^[^ ^247^ ^]^ and other conditions like cancer therapy.^[^ ^248^ ^]^ Conversely, cytosolic pH‐responsive nanocarriers can release the drug responsive to the intracellular pH, which emerged for the treatment of FNDs as follows.

Nanomedicine could be absorbed by cells via the endocytic pathway.^[^ ^249^ ^]^ However, it should escape endosomal trapping to release the payload into the cytoplasm. Particularly, nucleic acids such as siRNAs are vulnerable in acidic endosomal environments. To overcome this barrier, Guo et al. strategically designed a pH‐responsive triblock copolymer PEG‐b‐PAMA‐b‐P(C7A‐r‐DBA) (PACD) with an A‐B‐C configuration (Figure 12c).^[^ ^242^ ^]^ PACD consists of 1) a hydrophilic PEG block, 2) a block for binding siRNA, and 3) a pH‐responsive block. These PACD copolymers have the ability to autonomously assemble into nanoscale polymeric micelles. By merely mixing them, PACDs compact siRNAs into polyplexes. The significant alterations in PACD particle size observed under various pH conditions suggest that PACD remains stable and intact under neutral pH conditions. However, when exposed to an acidic environment akin to that of endosomes, characterized by a pH range of 6.5 to 5.5, PACD undergoes hydrolysis, facilitating the release of loaded siRNAs. The siRNA release data further demonstrated that PACD micelles displayed a swift and substantial release at pH 5.5, underscoring the role of the intracellular acidic environment within the cytoplasm in promoting efficient siRNA release inside cells. In contrast, at pH 7.4, siRNA release occurred more gradually and was sustained for up to Day 7. This provides additional confirmation of the robust electrostatic interaction between the polyplexes and siRNA within the physiological extracellular environment. In conclusion, the PACD/siRNA polyplexes demonstrate an outstanding ability to escape from intracellular endosomes, resulting in excellent gene silencing and the inhibition of retinal angiogenesis. Similarly, Tan et al. designed a versatile core‐shell system for precise intracellular delivery of the Flt23k plasmid, which encodes the VEGF‐binding domains of Flt1, to treat retinal diseases like AMD (Figure 12d).^[^ ^243^ ^]^ This system consisted of several components: 1) an amino acid‐functionalized dendrimer and nuclear localization signal core to enhance nucleic acid complexation, internalization, and nuclear localization, 2) a lipid bilayer inner shell comprising pH‐sensitive lipid DOPE/cholesteryl hemisuccinate (CHEMS) to improve endosomal escape, and 3) an outermost shell of HA‐1,2‐dioleoylphosphatidylethanolamine (DOPE) to enhance cellular uptake. In this nanoplatform, researchers opted for the utilization of the pH‐sensitive lipid CHEMS to construct a lipid bilayer, which exhibits fusogenic properties at the lower pH environment of endosomes, thereby facilitating efficient endosomal escape.^[^ ^250^ ^]^ Furthermore, the nanoplatform demonstrated safety profiles in both in vitro and in vivo settings, effectively suppressing VEGF expression in the hypoxia‐mimicking condition. Notably, in vitro assays displayed a substantial suppression of endothelial cell migration and tube formation. In vivo assessments underscored the successful delivery of this nanoplatform to the retina. Collectively, these findings highlight the promising potential of this nanoplatform for delivering gene materials to the retina, offering a prospective avenue for treating retinal diseases, including AMD.

### 4.4 Light‐Responsive Nanocarrier‐Based Nanomedicines

Light‐responsive nanocarriers can undergo deformation upon stimulation with a specific wavelength of light, leading to the release of the cargo, where the light‐responsive mechanisms include photocleavage, photoisomerization, reversible cross‐linking/de‐cross‐linking, photopolymerization, photosensitization, and photothermal reactions.^[^ ^251^ ^]^

**Figure 13:**
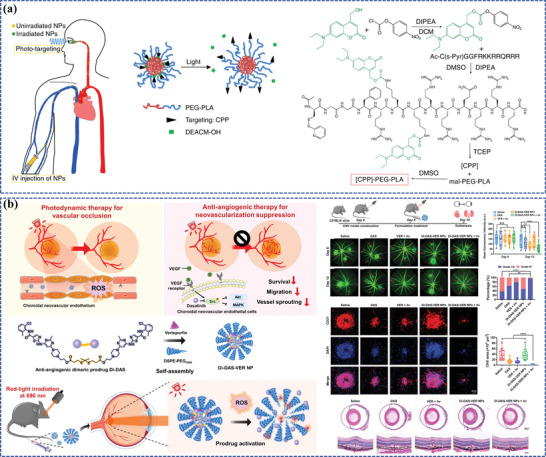
Light‐responsive nanocarrier‐based nanomedicines for the treatment of FNDs. a) Schematic of light‐triggered activation of the photo‐targeted nanoparticle and synthesis of the polymer chain functionalized with caged CPP. Reproduced under the terms of the CC‐BY.^[^ ^79b^ ^]^ Copyright 2019, The Authors, published by Springer Nature. b) Schematic illustration of the preparation of Di‐DAS‐VER, the combination of anti‐angiogenic therapy and PDT for wet AMD, and the CNV regression measured by FFA and staining images in the CNV mouse model. Reproduced under the terms of the CC‐BY.^[^ ^259^ ^]^ Copyright 2023, The Authors, published by Wiley‐VCH.

Among the light‐responsive mechanisms, photocleavage is considered the most utilized, which incorporates a photo‐cleavable group, such as o‐nitrobenzyl (ONB) coumarin, and their derivatives, into a polymer backbone, to regulate drug delivery.^[^ ^251c^ ^]^ The payload is then loaded into the matrix in a non‐covalent manner or linked to the polymer using a photolabile covalent linker, and subsequent irradiation of these polymers with light triggers the release of the drug.^[^ ^252^ ^]^ The ONB moiety serves as a light‐responsive protecting group and can respond to ultraviolet light in the range of 300–365 nm. Under these conditions, it undergoes decomposition to small molecules through quinone‐methide rearrangements, leading to rapid release of encapsulated therapeutic agents.^[^ ^253^ ^]^ On the basis of this theory, Fomina et al. designed a light‐triggered copolymer‐based nanosystem applying ONB moieties as ultraviolet‐labile photocages for self‐immolated polymers, whose degradation was triggered by ultraviolet radiation (330–450 nm).^[^ ^254^ ^]^ Then, in a following study, nanosystems loaded with nintedanib (BIBF), an anti‐angiogenic molecule, were intravitreally injected into rats before the laser‐induced formation of CNV^[^ ^255^ ^]^ and at the same time, the inhibitory effect on vessel growth was compared in rats treated with BIBF‐loaded PLGA nanoparticles (BIBF 1120), free drug, or saline. After 10 weeks of nanoparticles injection and 2 weeks after inducing CNV, the CNV lesion was significantly smaller in the eyes that received irradiation compared to other groups. The study has also shown that nanoparticles remained present in the vitreous chamber for up to 30 weeks after injection despite polymer hydrolysis. Coumarins are another functional groups conferring light‐responsive properties.^[^ ^256^ ^]^ Upon absorption of a photon by coumarin‐based conjugates, relaxation to the lowest excited singlet state occurs, followed by deactivation through fluorescence and non‐radiative processes. At the same time, heterolytic bond is cleaved, inducing the singlet ion pair consisting of the coumarin^+^ cation and the drug^−^ anion. Product formation occurs through two phases: first, solvent separation of the coumarin^+^ and drug^−^ anions, and then the reaction of the coumarin^+^ cation with water, resulting in the produce of coumarin‐OH and the release of the drug.^[^ ^257^ ^]^ On the basis of this theory, Wang et al. devised a sophisticated light‐responsive nanocarrier using a coumarin derivative‐modified PEG‐PLA block copolymer that could self‐assemble into nanoparticles (**Figure** **13**a).^[^ ^79b^ ^]^ This nanosystem adopted 7‐(diethylamino) coumarin‐4‐yl]methyl carboxyl (DEACM), a photocleavable caging group that is selected for its high photocleavage efficiency and relatively long (400 nm) absorption wavelength (low phototoxicity), and cell‐penetrating peptide (CPP), which enhanced cell uptake after the light‐responsive bond within DEACM was cleaved upon light irradiation. Upon exposure to light, the caging group underwent bond cleavage, leading to the removal of the protective group and allowing the peptide to bind to nearby cells. The DEACM‐CPP functionalized nanoparticles facilitated the targeted delivery of drugs to affected locations, thereby reducing off‐target drug delivery. Furthermore, In addition to the therapeutic advantages for FNDs, the coumarin‐based light‐responsive nanosystems were also capable of elevating the systemic administration efficiency of ocular targeted drug delivery. Likewise, in another study, green light‐responsive dicyanomethylene derivatives of coumarin were coupled with a trigonal core molecule, specifically tris(2‐aminoethyl) amine, which were capable of self‐assembling into nanocarriers, enabling drug accumulation in the fundus responsive to light.^[^ ^258^ ^]^

Photoisomerization refers to a reversible molecular transformation involving a constrained rotation point, commonly a double bond, triggered when exposed to visible or ultraviolet light. Compounds typically transition between trans and cis conformations within this framework, with azobenzenes and spiropyrans being extensively studied components for photoisomerization reactions to enable light‐driven drug release in a dynamic manner.^[^ ^260^ ^]^ Polymers incorporating azobenzenes, which consist of two phenyl rings linked by an azo group that undergoes a light‐responsive transformation from trans to cis conformation within the range of 320–350 nm, with the reverse process occurring between 400 and 450 nm, offer diverse and intriguing prospects due to their light‐sensitive characteristics.^[^ ^261^ ^]^ A self‐assembled cationic nanovesicle was generated from a cationic azobenzene derivative and the anionic surfactant sodium dodecyl sulfate in an aqueous solution, followed by loading Rhodamine B (used as a model drug) within these nanovesicles.^[^ ^262^ ^]^ Subsequently, this formulation was injected intravitreally into rats to evaluate its drug delivery performance, and UV irradiation led to a significant surge in rhodamine B release within the retinas compared to non‐irradiated samples. Furthermore, the effectiveness of these nanovesicles in facilitating retinal drug delivery was demonstrated by fluorescence images of retinal sections, which also showcased their capacities to maintain elevated drug concentrations for an extended period. Furthermore, explorations into azobenzene photoswitches have focused on their abilities to recover light sensitivity in retinal neurons affected by disorders like photoreceptor degeneration.^[^ ^262^ ^]^ A particularly effective photoswitch, BENAQ, composed of an azobenzene structure with a quaternary ammonium (QA) group and a benzylethylamine moiety, can penetrate through retinal ganglion cells' plasma membranes and inhibit their internal voltage‐gated ion channels. The photoisomerization of BENAQ from its trans to *cis* state could eliminate this inhibition, enabling the retinal ganglion cells to depolarize and trigger action potentials, where the transformation of BENAQ could be induced by visible light and could be reversible in dark conditions.^[^ ^263^ ^]^ Additionally, the incorporation of BENAQ into cyclodextrins could address the primary challenge associated with BENAQ delivery, which tends to precipitate near the injection site, leading to uneven photosensitization with a transient therapeutic window of seven days for vision restoration.^[^ ^264^ ^]^ The formation of a complex with sulfobutylether β‐cyclodextrin (SBE‐CD) enhanced BENAQ's solubility and extended its light‐responsive effect to a half‐life of 31 days, which showcased a compelling application of supramolecular chemistry in intraocular drug delivery. Spiropyran is another light‐triggered photoisomerization component. The reversible isomerization of spiropyran results in a noticeable color alteration attributed to the distinct molecular characteristics of the molecule's two isomers, where spiropyran is the more stable form representing the closed‐ring isomer.^[^ ^251c^ ^]^ As a promising light‐triggered photoisomerization component, more research is needed to advance spiropyran‐based light‐responsive nanocarriers for managing ocular diseases.

Photopolymerization finds extensive application for developing polymer‐based biomaterials, usually accomplished by exposing monomers to light with photoinitiators.^[^ ^265^ ^]^ Ultraviolet and halogen light (400–520 nm) are the typical light sources, and photoinitiators are the essential compounds that initiate the polymerization process by generating reactive molecules when exposed to light.^[^ ^266^ ^]^ Photoinitiators, including irgacure 2959, eosin, and 1‐cyclohexyl phenyl ketone, can prompt the formation of reactive species (anionic, cationic, or free radicals), either through light or heat, leading to a polymerization chain reaction where radicals interact with monomers and then produce more radicals for further reactions.^[^ ^267^ ^]^ Therefore, the choice of monomers is instrumental in tailoring features like mechanical attributes, cellular adhesion, and biodegradability. The precision control provided by photopolymerization, along with its rapid curing rates at normal temperatures, makes it suitable for non‐invasive or minimally invasive techniques.^[^ ^251^, ^268^ ^]^ One example is that a study demonstrated the light‐induced crosslinking of PCL dimethacrylate and glycol methacrylate to control the delivery of bevacizumab, where the transparency of the cornea and the crystalline lens facilitates non‐invasive light access to the ocular posterior segment.^[^ ^269^ ^]^ In a study by Bisht et al., peptide‐loaded PLGA nanoparticles were incorporated into methacrylated alginate, and light was utilized to initiate gelation for vitreous body implantation.^[^ ^270^ ^]^

PDT involves using a specific wavelength of light to activate a photosensitizer and oxygen, leading to the generation of ROS, followed by resulting in oxidative damage of cellular constituents, triggering apoptosis or pyroptosis.^[^ ^271^ ^]^ PDT has undergone extensive study and development as an anti‐cancer therapy,^[^ ^272^ ^]^ and has been emphasized as an alternative antimicrobial approach in the emerging era of antibiotic resistance.^[^ ^273^ ^]^ Particularly, PDT is a therapeutic strategy employed for FNDs, which operates laser to activate a photosensitizer, resulting in the apoptosis of vascular endothelial cells, thereby reducing leakage and occluding fundus neovascularization.^[^ ^274^ ^]^ Although PDT is widely used to treat CNV due to the eye's excellent light transmittance, it can cause damage to surrounding tissues and worsen the microenvironment, leading to increased hypoxia, inflammation, and secondary neovascularization. Intriguingly, utilizing PDT both for more precisely occluding fundus neovascularization and triggering the release of drugs thus achieved a “one stone, two birds” effect, Xu et al. enabled effective intravesical CNV treatment by incorporating a photoactivation process with combination therapy into a simple nanosystem (Figure 13b).^[^ ^259^ ^]^ To illustrate, photoactivatable prodrug‐based nanoparticles, Di‐DAS‐VER, were engineered by co‐assembling a ROS‐responsive dimeric dasatinib prodrug (Di‐DAS), verteporfin, and the amphiphilic lipid DSPE‐PEG2000. Upon exposure to red light at 690 nm, verteporfin could generate ROS, inducing the photodynamic effect. Simultaneously, this light exposure triggered the cleavage of the dimeric nano‐prodrug, leading to a sequential release of dasatinib (DAS). In mice models, systemic administration of this nano‐prodrug followed by red‐light irradiation on CNV eyes resulted in ROS overproduction and intraocular release of DAS. This dual mechanism enabled PDT‐induced vascular occlusion and DAS‐induced CNV inhibition. Importantly, systemic adverse reactions were minimized through intravenous administration since the prodrug remained inactive in normal tissues without exposure to red light.

PTT is a therapeutic approach that utilizes the photothermal conversion effect of photothermal transduction agents, which has been developed for cancer therapy,^[^ ^275^ ^]^ bacteria‐induced infections,^[^ ^276^ ^]^ and posterior capsule opacification.^[^ ^277^ ^]^ Intriguingly, Au NPs have the unique ability to efficiently absorb light energy from UV, visible, and NIR sources and convert it into heat within picoseconds, which occurs when the wavelengths of the incoming light align with the localized surface plasmon resonance absorption bands of the Au NPs.^[^ ^278^ ^]^ Consequently, the absorbed energy generates a high‐energy plasmonic electron gas through the collective oscillation of conduction band electrons. The excess energy is rapidly dissipated as heat, exchanging thermal energy with the particle's surrounding environment. Therefore, light‐absorbing materials like Au NPs incorporated into DDSs have the ability to absorb incident photons and convert them into thermal energy, causing the DDS to rupture and leading to the release of the payload.^[^ ^279^ ^]^ Similarly, the use of NIR fluorescent dyes has been explored in ocular drug delivery. These dyes emit light in the wavelength range of 740–1700 nm, offering reduced adverse effects, high photothermal transformation efficiency, as well as long absorption wavelengths. For instance, trihexylsilyloxide has been employed as a photothermal agent to activate light‐responsive lipid‐based nanocarriers, aiming to develop a potential nanomedicine for the treatment of AMD.^[^ ^280^ ^]^ Likewise, HA‐coated liposomes were utilized to encapsulate ICG as a light‐responsive component for the controlled intravitreal delivery of calcein, and the prolonged irradiation periods resulted in increased release of calcein from the liposomes.^[^ ^281^ ^]^ In another study, ICG was encapsulated within liposomes to be delivered to the retina for PTT, and the liposomes effectively addressed the challenges commonly associated with ICG delivery, such as quenching, aggregation, and instability.^[^ ^282^ ^]^

As has been noted, most light‐responsive nanocarrier‐based nanomedicines are in the proof‐of‐concept stage still. Photochemical triggering typically leads to an irreversible change in the nanosystem, making it suitable for applications requiring a single burst of drug release rather than gradual and repeated pulses. This approach is advantageous when the goal is to destroy the target tissue, such as in the treatment of cancer cells and AMD. However, it may not be an ideal strategy when the objective is solely drug delivery and targeting without tissue destruction. Although light‐responsive nanocarriers have shown huge potential as promising clinical nanoplatforms that would require less frequent IVTs and improve patient compliance, crucial evaluations need to be exerted to ensure the safety and effectiveness of the platforms and are necessary to establish their suitability for clinical applications.

### 4.5 Ultrasound‐Responsive Nanocarrier‐Based Nanomedicines

The application of ultrasonic energy to initiate the payload release typically involves two primary pathways: a) the disruption of the chemical bonds that connect the drug and its carrier and b) the obliteration of the carrier itself, subsequently releasing the payload.^[^ ^283^ ^]^ In the first pathway, when the ultrasonic wave disrupts the chemical bonds, it may cause an imbalance between the molecules’ hydrophilic components and their hydrophobic counterparts. This disequilibrium can lead to the collapse of the delivery system, ensuring that the payload is released.^[^ ^283b^ ^]^ In the second pathway, the carriers can be obliterated under the influence of ultrasound, which provokes cavitation or the conversion of energy into heat and motion.^[^ ^226^, ^284^ ^]^ The cavitation, defined by a precipitous plunge in liquid pressure that spawns minuscule cavities, plays a pivotal role in ultrasonically triggered payload release and eclipses the thermal effects in terms of importance.^[^ ^284a^ ^]^ Typically, payload release involves two specific forms of cavitation: the stable and the transient varieties.^[^ ^283b^ ^]^ Stable cavitation involves the oscillation of bubbles in response to low‐frequency ultrasound, leading to changes in bubble size and the formation of liquid micro‐streams surrounding the bubbles, which then exert shearing forces that trigger the release of payload from its carrier. In the meantime, stable cavitation also has the added benefit of acoustic perforation, which represents the occurrence of reversible pores on the surfaces of cell and capillary membranes, thus augmenting the penetrability of these membranes.^[^ ^284a^ ^]^ In instances of higher ultrasonic intensity, however, bubbles can oscillate in an unstable manner, growing to an unsustainable size before collapsing, a process known as transient cavitation.^[^ ^283a^ ^]^ The collapse of these bubbles is capable of emitting powerful shockwaves and jets that not only tear through the drug carrier to release the payload but also cause acoustic perforation and generate significant amounts of heat.^[^ ^231^ ^]^

Nabilim et al. revealed a significant enhancement of corneal penetrability for sodium fluorescein and dexamethasone in vitro during ultrasonic experiments. Under ultrasound exposure, sodium fluorescein's corneal penetrability increased by 46–126%, while that of dexamethasone increased by 32–109%. This increase was most prominent at an ultrasound frequency of 400 kHz.^[^ ^285^ ^]^ Complementing these findings, the in vivo studies by Suen et al. demonstrated that ultrasonic waves applied to the sclera considerably augmented the delivery of macromolecules like dextran to the vitreous body.^[^ ^286^ ^]^ In cases without ultrasonic treatment, dextran was not detectable in the vitreous, but after a single ultrasonic session, the concentration of fluorescein‐tagged dextran reached 0.022 µg/g and increased 70‐fold following three ultrasonic sessions. Further assessments of retinal structure and function confirmed that ultrasonic application did not cause any harm. This underscores the utility of ultrasound in safely and effectively facilitating drug delivery across the ocular barriers.^[^ ^226a^ ^]^

Zhou et al. further substantiated the benefits of the ultrasound‐microbubble technique in enhancing nucleic acid transfection efficiency for PEDF and suppressing the development of CNV.^[^ ^287^ ^]^ Over a 28‐day treatment period, this method showed a significant improvement in PEDF gene transfection compared to the liposome‐based approach. Likewise, Li et al. observed that the destruction of microbubbles in the process of rAAV2‐CMV‐EGFP transfection, initiated by ultrasonic wave, significantly expedited and increased the specific expression of targeted transgene within the retina.^[^ ^288^ ^]^ These findings collectively highlight the potential of ultrasound as a promising and safe technique for facilitating therapeutic agent delivery across ocular barriers and promoting gene transfection in the eye.

Thakur et al. developed highly echogenic ultrasound‐responsive nanobubbles focusing on size optimization for enhanced stability. Their findings demonstrated that compared to systems without ultrasound and nanobubbles, the application of specific ultrasonic protocols, namely 1 W cm^−2^ for 20 s and 0.5 W cm^−2^ for 30 s, significantly and rapidly improved the internalization of rabbit immunoglobulin G (IgG) within MIO‐M1 (human Müller stem cell line) and ARPE‐19 cells in vitro.^[^ ^289^ ^]^ In further ex vivo studies, the same team evaluated the effectiveness of the nanobubbles in transporting drugs intravitreally, using both ex vivo bovine and porcine eye models.^[^ ^290^ ^]^ The study employed bovine eye models to reveal that, in the absence of ultrasound, less than 10% of the nanobubbles were present in the ocular posterior segment. However, after a single corneal ultrasound cycle, ≈28.6% ± 8.5% of the nanobubbles were transferred to the ocular posterior segment, leading to a significant enhancement in their displacement (*p* < 0.001). Following three ultrasound cycles, the nanobubbles in the posterior vitreous body constituted ≈47.8% ± 15.2%. Additionally, ultrasound exhibited effective directionality in isolated porcine eye models as nanobubbles remained below 10% in the non‐acting direction after ultrasound application. This highlights the precise control offered by ultrasound, which, owing to its conversion into thermal energy, requires careful regulation when applied to treat ocular conditions. In the same study, the researchers employed a 60 s ultrasonic protocol with stimulus‐free intervals to mitigate potential thermal damage to ocular tissues and further enhance the targeted migration of nanobubbles. This approach underscores ultrasound's significant potential in expediting and directing drug delivery to the retina, particularly when rapid and precise intervention is warranted.

## 5 Conclusions and Prospects

FNDs are serious ocular diseases that impair vision. The current treatment of anti‐VEGF IVT has significant defects, including incomplete response, complications including eye pain, inflammation, retinal detachment, and macular dysfunction. At the same time, due to numerous limitations in ocular posterior drug delivery barriers, conventional therapies cannot offer comprehensive therapeutic solutions for ocular fundus diseases, posing significant clinical challenges. The ongoing development of nanomedicine holds great promise for FND management, providing higher ocular posterior drug delivery efficiency and effective treatment for FNDs. In terms of ocular posterior drug delivery, nanocarriers can effectively traverse the ocular barriers and make up slow‐release formulations to decrease the dosing interval, with the potential to target specific cell types to minimize off‐target effects on unaffected retinal cells. With respect to therapeutic effects, nanomedicine, especially smart nanocarrier‐based nanomedicines, can enhance the anti‐angiogenic effect, reduce side effects, and fulfill precise drug release control responsive to various stimuli. This targeted and personalized approach has the potential to revolutionize the management of FNDs.

Apart from the forms of smart nanocarriers mentioned above, there is a wide range of various types of stimuli‐responsive nanocarriers available that are utilized in other diseases. When developing stimuli‐responsive nanocarriers, endogenous variations are attractive targets due to their frequent association with significant markers for various types of diseases.^[^ ^26a^ ^]^ Meanwhile, other typical endogenous stimuli include glucose, ATP, hypoxia, nucleic acids, as well as mechanical cues.^[^ ^291^ ^]^ For instance, hypoxia is a common characteristic observed in various disease conditions, particularly in cancer and FNDs.^[^ ^292^ ^]^ Thus, hypoxia‐responsive nanomedicine that may deliver and release cargo responding to hypoxic conditions has been highly sought after for precision medicine of cancer therapy,^[^ ^293^ ^]^ which is enlightening for FND therapy. Meanwhile, to achieve on‐demand drug release, the ATP‐controlled DDSs usually utilize ATP‐targeted aptamers as mediums.^[^ ^294^ ^]^ Specifically, ATP can induce conformational changes that generate structure‐disorder forces or competitively bind to drug‐loading sites to initiate drug release.^[^ ^295^ ^]^ Therefore, hypoxia and ATP are the other two types of stimuli with huge potential to develop various smart nanocarrier‐based nanomedicines.

Looking ahead, increased attention should be directed toward the development of innovative and noninvasive nanomedicines that can effectively surmount ocular barriers, extend drug release duration, and maintain therapeutic concentration at targeted lesion sites. This requires the optimization of various nanomedicine properties, including size, zeta potential, biocompatibility, stability, pH, surface tension, and osmotic pressure. Concurrently, more extensive in vitro and in vivo experiments should be conducted, animal models that closely resemble FNDs need to be established, and the methods for evaluating therapeutic efficacy should be enhanced to provide more accurate predictions regarding the safety and effectiveness of nanomedicine. Furthermore, exploring novel nanomedicines such as dual‐ or multi‐responsive nanocarrier‐based nanomedicines, gene therapy‐based nanomedicines, exosomes, and nanomedicine‐mediated tissue engineering holds great promise for advancing FND management.

## Conflict of Interest

The authors declare no conflict of interest.

## Author Contributions

Y.Z., M.X., and W.S. contributed equally to this work. Y.Z., M.X., and W.S. performed conceptualization, investigation, and original draft writing. Y.X., A.S., and K.Y. performed review and editing. P.X. performed review, editing, and acquired funding. H.H. and J.Y. provided guidance, supervision, funding acquisition, review, and revision. All authors approved the final manuscript and publication.
